# Elucidation of the calcineurin-Crz1 stress response transcriptional network in the human fungal pathogen *Cryptococcus neoformans*

**DOI:** 10.1371/journal.pgen.1006667

**Published:** 2017-04-04

**Authors:** Eve W. L. Chow, Shelly A. Clancey, R. Blake Billmyre, Anna Floyd Averette, Joshua A. Granek, Piotr Mieczkowski, Maria E. Cardenas, Joseph Heitman

**Affiliations:** 1 Department of Molecular Genetics and Microbiology, Duke University Medical Center, Durham, North Carolina, United States of America; 2 Department of Biostatistics and Bioinformatics, Duke University Medical Center, Durham, North Carolina, United States of America; 3 Duke Center for the Genomics of Microbial Systems, Duke University Medical Center, Durham, North Carolina, United States of America; 4 High-Throughput Sequencing Facility, School of Medicine, University of North Carolina, Chapel Hill, North Carolina, United States of America; University of Melbourne, AUSTRALIA

## Abstract

Calcineurin is a highly conserved Ca^2+^/calmodulin-dependent serine/threonine-specific protein phosphatase that orchestrates cellular Ca^2+^ signaling responses. In *Cryptococcus neoformans*, calcineurin is activated by multiple stresses including high temperature, and is essential for stress adaptation and virulence. The transcription factor Crz1 is a major calcineurin effector in *Saccharomyces cerevisiae* and other fungi. Calcineurin dephosphorylates Crz1, thereby enabling Crz1 nuclear translocation and transcription of target genes. Here we show that loss of Crz1 confers phenotypes intermediate between wild-type and calcineurin mutants, and demonstrate that deletion of the calcineurin docking domain results in the inability of Crz1 to translocate into the nucleus under thermal stress. RNA-sequencing revealed 102 genes that are regulated in a calcineurin-Crz1-dependent manner at 37°C. The majority of genes were down-regulated in *cna1*Δ and *crz1*Δ mutants, indicating these genes are normally activated by the calcineurin-Crz1 pathway at high temperature. About 58% of calcineurin-Crz1 target genes have unknown functions, while genes with known or predicted functions are involved in cell wall remodeling, calcium transport, and pheromone production. We identified three calcineurin-dependent response element motifs within the promoter regions of calcineurin-Crz1 target genes, and show that Crz1 binding to target gene promoters is increased upon thermal stress in a calcineurin-dependent fashion. Additionally, we found a large set of genes independently regulated by calcineurin, and Crz1 regulates 59 genes independently of calcineurin. Given the intermediate *crz1*Δ mutant phenotype, and our recent evidence for a calcineurin regulatory network impacting mRNA in P-bodies and stress granules independently of Crz1, calcineurin likely acts on factors beyond Crz1 that govern mRNA expression/stability to operate a branched transcriptional/post-transcriptional stress response network necessary for fungal virulence. Taken together, our findings reveal the core calcineurin-Crz1 stress response cascade is maintained from ascomycetes to a pathogenic basidiomycete fungus, but its output in *C*. *neoformans* appears to be adapted to promote fungal virulence.

## Author summary

The ubquitiously conserved serine/threonine-specific protein phosphatase calcineurin is crucial for virulence of several opportunistic human fungal pathogens including *Candida albicans*, *Aspergillus fumigatus*, and *Cryptococcus neoformans*. We show that Crz1 acts downstream of calcineurin, to 1) govern expression of genes involved in cell wall integrity, and calcium and small molecule transport, and 2) contribute to stress survival and virulence of *C*. *neoformans*. Our studies reveal that calcineurin also controls mRNA expression levels of other genes independently of Crz1. We propose that calcineurin operates in a branched signal transduction cascade controlling targets at both the transcriptional and post-transcriptional levels.

## Introduction

Ca^2+^ signaling cascades are employed by eukaryotic cells to control gene expression and other cellular processes. Calcineurin is a highly conserved Ca^2+^/calmodulin-dependent serine/threonine-specific protein phosphatase that plays a central role in orchestrating cellular responses to Ca^2+^ signaling in fungi and mammals [1]. Calcineurin consists of two subunits: a catalytic A subunit (CNA) and a regulatory B subunit (CNB). Calcineurin activity is inhibited by the immunosuppressive, antifungal drugs tacrolimus (FK506) and cyclosporine A (CsA), which bind to the immunophilins FKBP12 and cyclophilin A, respectively [2, 3]. The cyclophilin A-CsA and FKBP12-FK506 complexes bind to calcineurin and inhibit its phosphatase activity by blocking substrate access to the calcineurin active site [2, 4]. In mammalian systems, calcineurin dephosphorylates and activates the NF-AT family of transcription factors [5]. In resting T-cells, NF-AT accumulates in the cytosol in a phosphorylated form. Antigen presentation to the T-cell receptor elicits a Ca^2+^ signal, and calcineurin dephosphorylates NF-AT to result in the translocation of dephosphorylated NF-AT into the nucleus where it induces the expression of cytokine genes.

In the model budding yeast *Saccharomyces cerevisiae*, calcineurin is not essential for growth under standard culture conditions. However its function is necessary for maintaining cell viability under specific environmental stress conditions such as the presence of high concentrations of various cations including calcium (Ca^2+^), sodium (Na^+^), and lithum (Li^+^) ions, or endoplasmic reticulum stress [6–10]. The transcription factor Crz1 (Calcineurin Responsive Zinc Finger 1) is a phosphoprotein and substrate of calcineurin, functioning downstream as the major effector of calcineurin in *S*. *cerevisiae* [11]. Disruption of the *CRZ1* gene results in similar but less severe phenotypes compared to calcineurin mutants, while overexpression of *CRZ1* suppresses calcineurin mutant phenotypes [12]. Similar to the mammalian model, calcineurin controls the activity of Crz1 by regulating its subcellular localization. Under standard conditions, Crz1 is phosphorylated and localized in the cytoplasm. Upon activation, calcineurin dephosphorylates Crz1, resulting in the rapid translocation of Crz1 into the nucleus [13].

The calcineurin-Crz1 signaling pathway plays important roles in stress responses and virulence and is conserved across various pathogenic fungi of both plants and mammals, including *Candida* species, *Aspergillus fumigatus*, *Magnaporthe oryzae*, and *Botrytis cinerea* [14–18]. In *A*. *fumigatus*, a filamentous human pathogenic fungus, calcineurin is required for regular septation and hyphal growth; *cnaA*Δ mutants form compact colonies with blunt hyphae with irregular branching, while *cnaB*Δ and *cnaA*Δ *cnaB*Δ double mutant strains form hyphae with impaired tip branching [19]. Loss of CrzA also results in reduced asexual sporulation and conidiation, and acute sensitivity to ionic stresses and heat-mediated killing [16, 20]. However, hyphal morphology defects are less severe in the *crzA*Δ mutant than the *cnaA*Δ mutant. Virulence of the *crzA*Δ mutant is greatly attenuated due to increased susceptibility to ionic stresses and reduced hyphal growth and impaired tissue penetration. Similarly, in *B*. *cinerea*, a filamentous plant pathogenic fungus, loss of Crz1 impairs vegetative growth and alters hyphal morphology, significantly reduces conidiation and sclerotium formation, and attenuates virulence [15]. In the human pathogenic *Candida* species, *C*. *albicans* and *C*. *glabrata*, *crz1*Δ mutants display less severe phenotypes than calcineurin mutants. In *C*. *glabrata*, loss of calcineurin renders cells sensitive to high temperature (40°C) and cell wall stress, but the *crz1*Δ mutant displays an intermediate phenotype between wild-type and calcineurin mutants [17, 21]. In *C*. *glabrata* and *C*. *albicans*, calcineurin, but not Crz1 is required for growth in the presence of serum [18, 21]. In *C*. *albicans*, loss of Crz1 causes increased azole and serum susceptibility, but only modestly attenuates virulence and confers an intermediate phenotype in response to ionic stresses compared to calcineurin mutants [17, 18, 22]. Comparison of calcineurin-Crz1-dependent genes identified in *M*. *oryzae* to those identified in *S*. *cerevisiae* and *A*. *fumigatus* revealed that only a small subset of 9 genes were shared in common suggesting that the calcineurin-Crz1 pathway is plastic, and that gain or loss of target genes is species-specific and may be attributed to evolution and transcriptional rewiring [11, 14, 23].

*Cryptococcus neoformans* is a globally distributed human fungal pathogen that causes meningoencephalitis in immunocompromised individuals, such as HIV/AIDS patients, with more than one million cases of cryptococcosis and ~620,000 mortalities reported annually [24]. In *C*. *neoformans*, calcineurin mutation results in loss of virulence [25, 26]. Calcineurin relocalizes to P-bodies and stress granules following thermal stress, and may therefore play novel post-transcriptional physiological roles [27]. Recent studies have implicated a candidate *Cryptococcus* Crz1 ortholog, albeit with conflicting conclusions [28–30]. Adler *et al*. first identified a gene (CNAG_00156) encoding a putative *CRZ1* ortholog, noting that it contains three Cys2-His2-type zinc finger domains in its C-terminal region, unlike the *S*. *cerevisiae* Crz1 that contains only two zinc finger domains. They found that *crz1*Δ mutants displayed phenotypes different from calcineurin mutants; notably the mutant was able to grow at 37°C, and displayed attenuated virulence in a murine infection model. The authors named the gene *SP1*, concluding that it was not a Crz1 ortholog and was instead homologous to the Sp1/KLF transcription factor family in metazoans that has three C-terminal Cys2-His2-type zinc finger domains [28–30]. In the second study, Lev *et al*. identified this same gene based on a similarity search using the zinc finger domains of *S*. *cerevisiae* Crz1. Employing the yeast two-hybrid assay, they found that the calcineurin catalytic A subunit interacted with the CNAG_00156 gene product and concluded that it indeed encodes an ortholog of *S*. *cerevisiae* Crz1. While they also found that the mutant strain displayed phenotypes that were different from calcineurin mutants, they did observe the translocation of Crz1 to the nucleus in a calcineurin-dependent fashion under thermal stress or in the presence of CaCl_2_ [29]. In the third study, Moranova *et al*. identified the same gene through a random insertional mutagenesis screen for genes responding to hypoxic stress, and reported that it was required for survival under limited aeration [30]. They concluded that it might be an ortholog of Crz1.

Although the importance of calcineurin in *C*. *neoformans* has been well established, and a candidate Crz1 ortholog identified, their downstream targets have yet to be elucidated. In a recent study, we demonstrated that upon thermal stress, Crz1 dephosphorylation, nuclear translocation and transcriptional activity is governed by calcineurin [31]. In this study, we show that calcineurin control of Crz1 is dependent upon integrity of the calcineurin docking domain of Crz1 in *C*. *neoformans*. We demonstrate that loss of Crz1 results in an intermediate phenotype compared to wild-type and calcineurin mutant strains. We elucidated the calcineurin-Crz1 signaling pathway by conducting RNA-sequencing, and found that the pathway controls genes that are involved in cell wall integrity, ion and small molecule transport, oxidation-reduction processes, and metabolism. Strikingly, the genes regulated by the calcineurin-Crz1 signaling pathway in *C*. *neoformans* under thermal stress are vastly different from those previously identified in *S*. *cerevisiae* and *A*. *fumigatus* under calcium or sodium stress [11, 23]. These results suggest that under different stress conditions, the calcineurin-Crz1 pathway regulates different gene sets, and that network re-adaptation occurred as the fungus evolved in its environmental niche. We also identified 393 genes that are regulated by calcineurin, independently of Crz1 and 59 genes that are regulated by Crz1 independently of calcineurin. By employing both MEME (Multiple Expectation maximization for Motif Elucidation) and DREME (Discriminative Regular Expression Motif Elicitation) analyses, we identified three putative motifs within the promoter regions of 81 out of the 102 genes regulated by the calcineurin-Crz1 pathway. We demonstrated that Crz1 binding to target gene promoters is increased upon thermal stress in a calcineurin-dependent fashion. Our results indicate that calcineurin acts on both Crz1 and other factors that govern mRNA expression or stability to operate in a branched transcriptional/post-transcriptional stress responsive network necessary for cryptococccal virulence.

## Results

### Deletion of the calcineurin docking motif affects Crz1 function in *C*. *neoformans*

The transcription factor Crz1 is a major downstream effector of calcineurin in various model and pathogenic fungi. Recent studies have identified CNAG_00156 as the putative *C*. *neoformans CRZ1* ortholog, but drew conflicting conclusions that suggested this gene is 1) the ortholog of Crz1, 2) not the ortholog of Crz1, or 3) might be the ortholog of Crz1 [28–30]. In our recent study, we identified CNAG_00156 as a substrate of calcineurin [31], and in this study reciprocal BLAST searches revealed that CNAG_00156 is an ortholog of *S*. *cerevisiae CRZ1* (**S1 Fig**). Similar results were obtained with Crz1 from *C*. *albicans* and *A*. *fumigatus* CrzA (**S1 Fig**). Phylogenetic analysis performed using the protein sequences of *Cryptococcus* Crz1, including the outgroup species *Cryptococcus amylolentus* and *Tsuchiyaea wingfieldii*, demonstrated that the Crz1 protein is highly divergent between different fungal species, but all feature at least two zinc finger domains at the C-terminus (**S1 Fig**).

**Fig 1:**
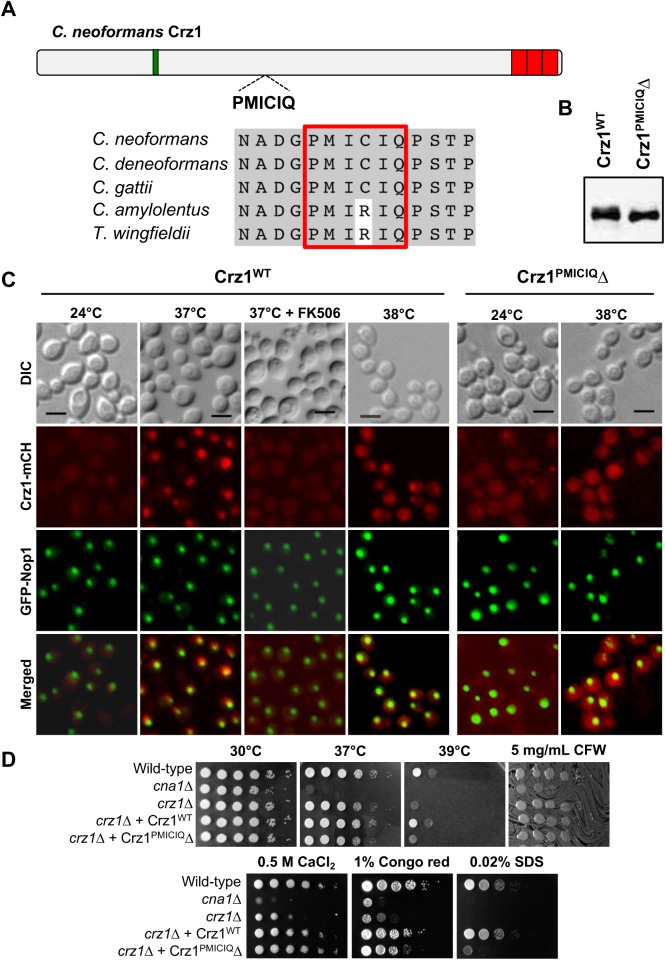
Deletion of the ^451^PMICIQ^456^ motif prevents translocation of Crz1 into the nucleus under thermal stress. **(A)** Schematic diagram showing the location of the candidate ^451^PMICIQ^456^ motif. The PMICIQ motif is conserved in the pathogenic species complex. The green box represents the PolyQ domain; red boxes represent the zinc finger domains. **(B)** Strains ECt172 (Crz1^WT^) and ECt386 (Crz1^PMICIQ^Δ) were grown overnight at 24°C, washed, and total cell lysates were extracted. Samples were resolved by SDS-PAGE and Western blotting was performed to verify expression of the Crz1^WT^ and the mutant Crz1^PMICIQ^Δ constructs. **(C)** The CRZ1^WT^-mCherry strain (ECt172) (Crz1-mCH) was grown at 24°C and then shifted to 37°C for 15 min. To verify that Crz1 translocation was due to calcineurin activity, FK506 (1 μg/ml) was added 30 min prior to the 37°C temperature shift. Strains ECt172 and ECt386 were grown at 24°C and then shifted to 38°C for 20 min. Cells were fixed with 4% formaldehyde for 15 min and washed, prior to imaging by direct fluorescence microscopy using the Zeiss Axioskop 2 Plus microscope. Scale bar = 5 μm. Note that GFP-Nop1 served as a nucleolar marker. **(D)** Wild-type (H99), *cna1*Δ (KK1), *crz1*Δ (AFA3-3) mutants, and strains ECt172 (*crz1*Δ + Crz1^WT^) and ECt375 (*crz1*Δ + Crz1^PMICIQ^Δ) were grown in YPD media, washed, and resuspended in PBS. Five 10-fold serial dilutions of each strain were spotted on YPD solid media, with the various additives as described and incubated at 30°C for 48 h, unless otherwise stated. Strains were incubated at 39°C for 72 h before imaging. CFW: calcofluor white.

In *S*. *cerevisiae* and *C*. *neoformans*, activated calcineurin dephosphorylates Crz1 leading to translocation of Crz1 into the nucleus and subsequent activation of stress genes [13]. In *S*. *cerevisiae*, the PIISIQ motif has been characterized as the calcineurin docking domain (CDD) in Crz1 [32]. In order to predict the CDD in *C*. *neoformans* Crz1, the PxIxIT consensus (P[^PG][IVFL][^PG][IVFL][TSHEDQNKR]) was queried against the CNAG_00156 ORF [31, 33]. We identified two candidate motifs in Crz1: ^451^PMICIQ^456^ and ^868^PALSIS^873^ (**Figs 1A** and **S3**). Comparison of the Crz1 protein sequences in the pathogenic *Cryptococcus* species complex, and two outgroup species, *C*. *amylolentus* and *T*. *wingfieldii*, revealed that the PALSIS motif is conserved across all of these species. However, the PMICIQ motif was conserved among the pathogenic species, while in the outgroup species, the cysteine has been substituted with arginine (**Fig 1A**).

To test if ^451^PMICIQ^456^ is a calcineurin docking domain, the motif was deleted from the wild-type Crz1, and both the Crz1^WT^ and the Crz1^PMICIQ^Δ mutant were fused with the mCherry protein and expressed in the *crz1*Δ mutant strain; expression of the Crz1-mutant construct was confirmed by Western blot (**Fig 1B**). We first verified if the calcineurin-dependent dephosphorylation of Crz1^WT^-mCherry expressed in the *crz1*Δ mutant strain results in its nuclear translocation. The strain expressing the Crz1^WT^-mCherry tagged protein (ECt172) was grown at 24°C, shifted to 37°C for 15 min, and then examined by direct fluorescence microscopy. At 24°C, the Crz1-mCherry signal was diffuse and evenly distributed throughout the cytosol (**Fig 1C**). Following incubation at 37°C for 15 min, Crz1-mCherry fluorescence was localized to the nucleus as evidenced by co-localization with the nucleolar marker protein GFP-Nop1 (**Fig 1C**). Furthermore, addition of the calcineurin inhibitor FK506 to the culture 30 min prior to the temperature shift prevented the translocation of Crz1 into the nucleus, consistent with Crz1 acting downstream of calcineurin (**S2 Fig**). Next, the strain expressing the Crz1^PMICIQ^Δ-mCherry tagged protein (ECt375) was grown at 24°C, shifted to 38°C for 20 min, and then examined by direct fluorescence microscopy. At 24°C, the mCherry signal was diffuse and evenly distributed throughout the cytosol (**Fig 1C**). Following incubation at 38°C for 20 min, mCherry fluorescence was still localized in the cytosol (**Fig 1C**).

**Fig 2:**
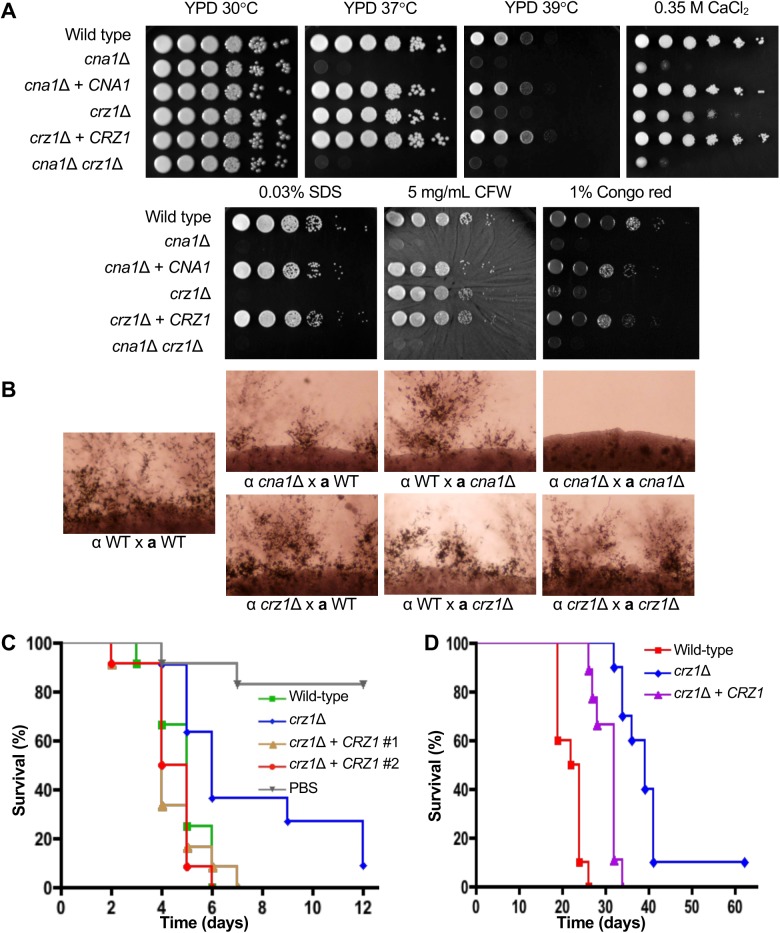
Deletion of *CRZ1* confers partial phenotypes in comparison to wild-type and *cna1*Δ mutant. **(A)** Wild-type (H99), *cna1*Δ (KK1), *crz1*Δ (AFA3-3) mutants and their respective complemented strains (KK5 and AFA3-3-17), and the *cna1*Δ *crz1*Δ double mutant strain (XW245) were grown in YPD media, washed, and resuspended in PBS. Five 10-fold serial dilutions of each strain were spotted on YPD solid media, with the various additives as listed and incubated at 30°C for 48 h, unless otherwise stated. Strains were incubated at 39°C for 72 h before imaging. CFW: calcofluor white. **(B)** Wild-type (H99 and KN99**a**), *cna1*Δ (KK1 and KK8), and *crz1*Δ (AFA3-3 and AFA1-4) strains of opposite mating type were grown in YPD media, washed, and resuspended in PBS. Equal cell numbers of opposite mating types were mixed and spotted on MS media. Mating assays were incubated at room temperature in the dark for 12 days and imaged. **(C)** 12 *G*. *mellonella* larvae per group were infected by injection with 4x10^3^ cells of wild-type (H99), *crz1*Δ (AFA3-3), or the *crz1*Δ + *CRZ1* complemented strains (AFA3-3-17 and ECt3). The larvae were incubated at 37°C and monitored daily. (H99 vs PBS, *p*-value <0.0001; AFA3-3 vs PBS, *p*-value = 0.0006; AFA3-3-17 vs PBS, *p*-value <0.0001; ECt3 vs PBS, *p*-value <0.0001; H99 vs AFA3-3, *p*-value = 0.0142; AFA3-3 vs AFA3-3-17, *p*-value = 0.0022; AFA3-3 vs ECt3, *p*-value = 0.0035; H99 vs AFA3-3-17, *p*-value = 0.2804; H99 vs ECt3, *p*-value = 0.5209) **(D)** Ten female BALB/c mice per group were infected through intranasal instillation with 1x10^6^ cells of wild-type (H99), *crz1*Δ (AFA3-3), and the *crz1*Δ + *CRZ1* complemented (AFA3-3-17) strains. The mice were monitored daily and sacrificed at predetermined clinical end points that are predictive of imminent mortality. (H99 vs AFA3-3, *p*-value <0.0001; AFA3-3 vs AFA3-3-17, *p*-value = 0.0002; H99 vs AFA3-3-17, *p*-value <0.0001)

Earlier studies have shown that *C*. *neoformans* calcineurin mutant strains are sensitive to high temperature and cell wall stresses [25, 26]. First, we determined if the deletion of *CRZ1* results in calcineurin-related phenotypes by assessing growth of the *crz1*Δ mutant at 37°C and 39°C and with a panel of different cell wall stresses. As previously reported, the *cna1*Δ mutant exhibits dramatic temperature sensitivity at 37°C and 39°C (**Fig 2A**). In contrast, the *crz1*Δ mutant displayed a modest temperature defect at 37°C compared to wild-type, and this was more pronounced at 39°C (**Fig 2A**). Similar to the *cna1*Δ mutant, the *crz1*Δ mutant displayed a growth defect on media containing 0.03% SDS. However, the *crz1*Δ mutant displayed an intermediate phenotype compared to the wild-type and *cna1*Δ mutants on media containing 0.35 M CaCl_2_ or 1% Congo red, and a very slight growth defect on media containing 5 mg/ml calcofluor white (**Fig 2A**). Complementation of the *crz1*Δ mutant with the wild-type *CRZ1* gene restored a wild-type phenotype under these stress conditions.

To determine if the deletion of PMICIQ motif confers any phenotypes, the *crz1*Δ + Crz1^WT^ and *crz1*Δ + Crz1^PMICIQ^Δ strains were assayed at 37°C and 39°C, in addition to a panel of different cell wall stresses (**Fig 1D**). We found that compared to the *crz1*Δ + Crz1^WT^ strain, the *crz1*Δ + Crz1^PMICIQ^Δ was growth impaired at 39°C, or in the presence of 0.02% SDS. The *crz1*Δ + Crz1^PMICIQ^Δ strain presented an intermediate phenotype between the wild-type and *crz1*Δ on media containing 1% Congo red (**Fig 1D**). In summary, our results show that deletion of the PMICIQ domain results in the inability of Crz1 to translocate to the nucleus and sensitivity to growth at high temperature, strongly suggesting that this domain is functional.

### The *crz1*Δ mutant displays an intermediate phenotype in comparison to wild-type and the calcineurin *cna1*Δ mutant

Calcineurin mutations impair hyphal elongation during sexual reproduction [25]. To determine if *crz1* mutations affect mating, unilateral and bilateral mating crosses of *crz1*Δ mutants of opposite mating types were conducted. Unlike the *cna1*Δ mutants, hyphal formation was not affected in the *crz1*Δ mutants in either unilateral or bilateral mating crosses, suggesting that either another target is involved or Crz1 and a second target of calcineurin are together redundant for the control of mating (**Fig 2B**). These phenotypic results support roles for Crz1 in cell wall integrity and calcium sequestration in the calcineurin signaling pathway, and suggest that calcineurin acts on factors other than Crz1 to govern thermotolerance and mating.

Calcineurin has been previously shown to be required for virulence and deletion of *CNA1* resulted in an avirulent phenotype [26]. In the *Galleria* infection model, we observed that loss of Crz1 resulted in reduced virulence in comparison to wild-type (*crz1*Δ mutant median survival = 6 days, WT median survival = 5 days; WT vs *crz1*Δ mutant *p*-value = 0.0142), which was restored by complementation of the *crz1*Δ mutant strain with the wild-type *CRZ1* gene (*crz1*Δ mutant vs *crz1*Δ + *CRZ1* complemented strain (ECt3 median survival = 4 days), *p*-value = 0.0035; *crz1*Δ mutant vs *crz1*Δ + *CRZ1* complemented strain (AFA3-3-17 median survival = 5 days), *p*-value = 0.0022) (**Fig 2C**). To determine if Crz1 is also required for the virulence of *C*. *neoformans* in a vertebrate model, animals were infected with the wild-type strain (H99), the *crz1*Δ mutant strain (AFA3-3), and the *crz1*Δ + *CRZ1* complemented strain (AFA3-3-17) (intranasal infection, 10^5^ CFUs). The *crz1*Δ mutant (median survival = 39 days) displayed attenuated virulence compared to wild-type (median survival = 23 days; *p*-value <0.0001) (**Fig 2D**). Complementation of the *crz1*Δ mutant with the wild-type *CRZ1* gene largely restored virulence (median survival = 32 days; *crz1*Δ + *CRZ1* vs wild-type *p-*value <0.0001; *crz1*Δ mutant vs *crz1*Δ + *CRZ1 p-*value = 0.0002) (**Fig 2D**).

We observed that the loss of *CRZ1* resulted in attenuated virulence in both infection models, which is in contrast to the fully avirulent phenotype displayed by the *cna1* calcineurin mutation (**Fig 2C and 2D**). The *crz1*Δ virulence defect was largely remediated and restored to wild-type when the *crz1*Δ mutant was complemented with the native *CRZ1* gene.

### Identification of calcineurin-Crz1 target genes by RNA-sequencing

A goal of this study was to identify the genomic targets of the calcineurin-Crz1-signaling pathway under high temperature stress. To this end, we performed RNA-sequencing of the wild-type strain, *cna1*Δ and *crz1*Δ mutant strains, and their respective complemented strains. In brief, strains were grown at 24°C and then shifted to 37°C for 1 hour, with three biological replicates. Direct polyA RNA sequencing was conducted and pairwise analyses of wild-type against the *cna1*Δ mutant and wild-type against the *crz1*Δ mutant were performed. Genes were regarded as differentially expressed if the expression ratio was altered ≥2-fold at a false discovery rate < 0.2.

**Fig 3:**
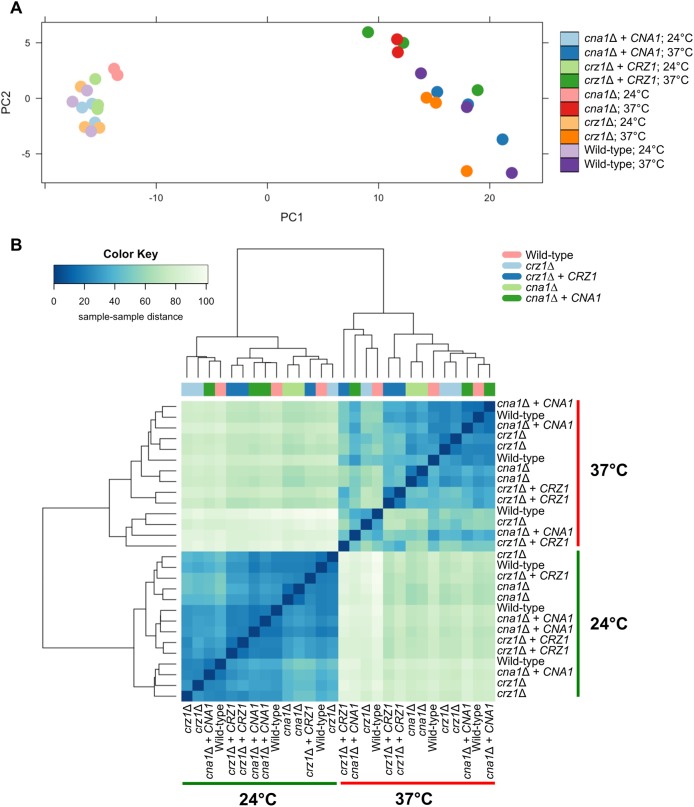
Expression dynamics of calcineurin and Crz1 target genes. **(A)** Principal component analysis (PCA) was performed to compare gene expression levels of the WT (H99), *cna1*Δ (KK1), *cna1*Δ + *CNA1* (KK5), *crz1*Δ (AFA3-3), and *crz1*Δ+ *CRZ1* (AFA3-3-17) strains grown at 24°C and 37°C. Lighter colors indicate the 24°C samples and the bright colors indicate the 37°C samples. Note that results for one *cna1*Δ 37°C biological replicate were not included in the analysis, due to sample contamination. **(B)** Hierarchical cluster analysis recapitulates the observation from the PCA. The heatmap indicates the pairwise distance between samples. The x-axis of the color key indicates the distances between the samples, with the darker blue signifying a higher similarity (i.e. small distance) between the samples, while the lighter blue signifies a lower similarity (i.e larger distance) between the samples. The color bar above the heatmap indicates the sample type. Gene expression levels at 24°C were different from the gene expression levels at 37°C. In general, gene expression of samples grown at 24°C showed less variability (dark blue). However, gene expression of samples grown at 37°C showed more variability (light blue).

Principle component analysis (PCA) and hierarchical clustering were used to explore the relationship in gene expression between samples (**Fig 3A and 3B)**. PCA demonstrated, unsurprisingly, that high-temperature stress has a larger effect on gene expression than either of the mutations. PC1 clearly distinguishes the samples grown at 37°C from the 24°C samples, which clustered to the right or left side of the graph respectively (**Fig 3A**). Moreover, the 24°C samples tightly clustered on PC1 and PC2, indicating much lower variability in gene expression under this growth condition, as is expected. In contrast, we observed greater variability in gene expression under temperature stress at 37°C for each of the strains analyzed (**Fig 3A**). A hierarchical cluster analysis was also preformed to verify the observations from the PCA. We observed the same clustering pattern, whereby gene expression patterns of strains grown at 24°C were more similar compared to each other than when compared to the gene expression of strains grown at 37°C (**Fig 3B**). The variability of gene expression at 37°C was again greater than that at 24°C, and the effect of the temperature shift is greater than the effect of the mutations (**Fig 3B**).

Quantitative real-time PCR was performed to validate the results obtained from RNA sequencing. Of the 22 genes analyzed, 20 genes showed very similar fold changes in expression compared to RNA-sequencing (**S3 Fig**). The magnitude of fold change in two genes (CNAG_02415 and CNAG_00025 (*VCX1*)) observed in the real-time PCR was higher than in the RNA-sequencing but still supported the relationships of the gene expression patterns among the isolates at 37°C. Only in one case (CNAG_07725 (*Rox1*)) were we not able to validate the RNA-sequencing results as the magnitude of fold change in wild-type at 37°C was lower by real-time PCR.

Pairwise analyses of wild-type against the *cna1*Δ mutant, and wild-type against the *crz1*Δ mutant at 24°C, showed that under non-stress inducing conditions, loss of *CNA1* and *CRZ1* did not have a significant impact on gene expression at a whole genome level. From the pairwise analysis of wild-type against the *cna1*Δ mutant, 32 genes were identified as being differentially expressed, and 50% of the genes were downregulated in the *cna1*Δ mutant, and the other half were up-regulated (**S1 Table**). Comparing the gene expression of the *crz1*Δ mutant against the wild-type, we found that only five genes were differentially expressed; of the five genes, four genes were also differentially expressed in the *cna1*Δ mutant (**S1 Table**).

**Fig 4:**
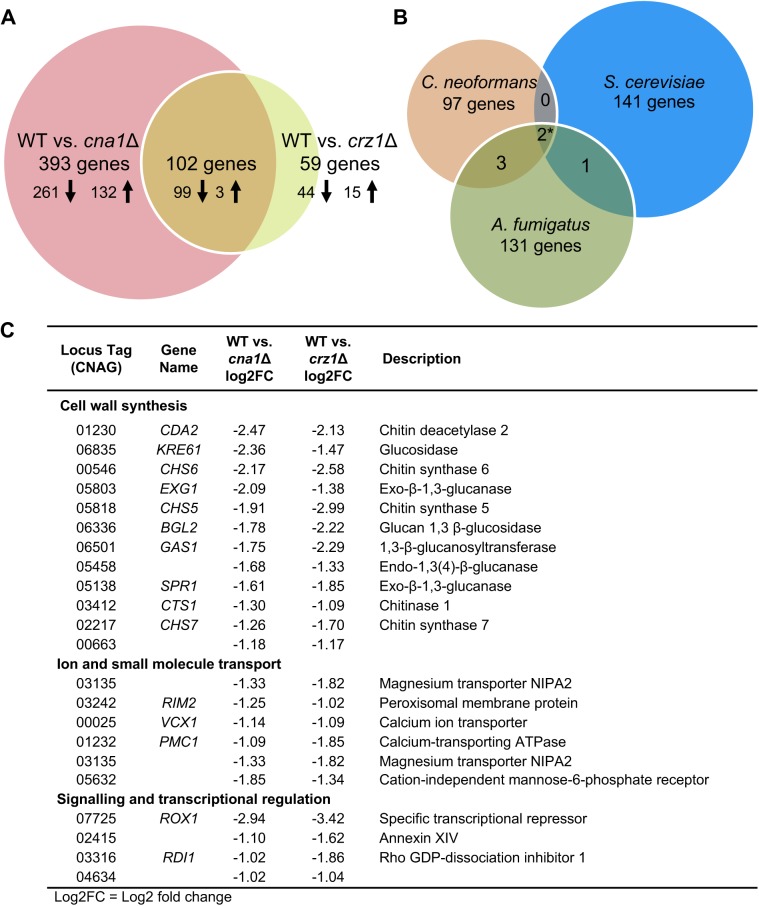
Gene suite regulated by the calcineurin-Crz1 pathway in *C*. *neoformans*. **(A)** Pairwise analyses of WT *vs*. *cna1*Δ and WT *vs*. *crz1*Δ gene sets were compared to determine the regulated genes. 102 genes from both gene sets have differential gene expression compared with the wild-type at 37°C and thus are controlled by both calcineurin and Crz1. 393 genes were differentially regulated by calcineurin, independently of Crz1, while 59 genes were regulated by Crz1 in a calcineurin-independent manner. **(B)** Comparison of the 102 genes against the known *S*. *cerevisiae* (144 genes) and *A*. *fumigatus* (137 genes) gene sets revealed that only two genes were shared in common between the three species (*Afu3g10690 and Afu7g01303 are both calcineurin dependent homologs of CNAG_0123 and YGL006W –refer to **S5 Table**). **(C)** Calcineurin-Crz1-dependent genes of known functions. Gene names and descriptions listed were identified using the FungiDB search portal; gene orthology was determined using the GO function. (Log2FC = Log2 Fold change)

At 37°C, 495 genes were identified as being differentially expressed and regulated in a calcineurin-dependent manner in the comparison between wild-type and the *cna1*Δ mutant. In contrast, only 161 genes were differentially expressed in the pairwise analysis of wild-type against the *crz1*Δ mutant at 37°C (**Fig 4A**).

To elucidate gene networks that are regulated by the calcineurin-Crz1 signaling pathway, the gene sets obtained from the pairwise analyses were compared. From the comparison of the 37°C gene sets, 102 genes were regulated in a calcineurin-Crz1 dependent manner (**Fig 4A**). 99 genes (97.1%) were down regulated in both the *cna1*Δ and *crz1*Δ mutants, indicating that these genes are normally positively activated by the calcineurin-Crz1-signaling pathway under thermal stress.

A majority of the genes (393 genes, 80%) identified as being calcineurin-dependent were directly or indirectly regulated independently of Crz1 (**Fig 4A** and **S2 Table**). 261 genes (66%) were down-regulated and 132 genes (34%) were up-regulated in the *cna1*Δ mutant, suggesting that calcineurin exerts a positive regulation of genes under thermal stress. Just over half of the genes (199 genes, 51%) encoded proteins of unknown function. 194 genes belonged to ascribed functional categories including: oxidation-reduction processes (35 genes, 18%), membrane transport including the ammonia permeases Amt1 and Amt2 and the inositol transporters Itr4 and Itr6 (32 genes, 8%), carbohydrate metabolism (20 genes, 5%), signaling and transcriptional regulation (14 genes, 3.5%), DNA replication (11 genes, 2.8%), sterol biosynthesis (5 genes, 1.3%), stress response (4 genes, 1%), and the remaining 73 gene products (18.6%) were involved in other metabolic and biological processes (**S2 Table**).

We identified a smaller subset of genes (59 genes) as being regulated by Crz1, independently of calcineurin, under thermal stress (**S3 Table**). 43 genes (73%) were down-regulated in the *crz1*Δ mutant, indicating that these genes are normally activated by Crz1 under thermal stress, and 16 genes (27%) were up-regulated. A majority of the genes (43 genes; 73%) encoded proteins of unknown functions. The remaining 16 genes had ascribed functional categories including: cell wall integrity (*FKS1* and *CHS8*), membrane transport (6 genes, 10%), oxidation-reduction processes (4 genes, 6.8%), carbohydrate metabolism (4 genes, 6.8%), protein kinases (3 genes, 5%), encoded vesicle-mediated transport (2 genes, 3.4%), and other metabolic and biological processes (5 genes, 8.5%) (**S3 Table**).

Of the 102 genes that are controlled by the calcineurin-Crz1 pathway, 64 (62.75%) are genes of unknown functions (**S4 Table**). Consistent with the hypothesis that Crz1 acts downstream of calcineurin to regulate cell wall integrity based on the phenotypic assays, 11% of the genes encode proteins that contribute to cell wall integrity/maintenance; examples of these include: *CHS7*, *CHS6*, *CHS5*, *KRE6*, *CDA2*, *CTS2*, *EXG1*, and *BGL2* (**Fig 4C**). We also identified genes involved in membrane transport (*VCX1*, *PMC1*, and *RIM1*), melanin production (*LAC2*), signaling and transcription (*ROX1*), and amino acid and carbohydrate metabolism (**Fig 4C**).

### Calcineurin-Crz1-signaling pathway has been adapted in *C*. *neoformans* to enable growth at 37°C

In *S*. *cerevisiae*, 163 calcineurin-Crz1-dependent genes were identified in a genome wide-profiling study under Ca^2+^ and Na^+^ stresses, including genes involved in ion homeostasis and cell wall integrity [11]. Here we focused on those that were calcium regulated (153 genes), and also excluded nine that were hypothetical genes, and from the remaining 144 genes we found that 79 have an ortholog in *C*. *neoformans*. Interestingly, when we compared the 102 calcineurin-Crz1-dependent genes identified in our study with the *S*. *cerevisiae* calcineurin-Crz1-dependent genes identified upon calcineurin stimulation by high Ca^2+^ stress, we found that while 30 genes have an ortholog in *S*. *cerevisiae*, only two genes (CNAG_01232 *PMC1* and CNAG_02217 *CHS7*) have a yeast ortholog that was also regulated by the calcineurin-Crz1 pathway (**Fig 4B** and **S5 Table**). Both genes were downregulated in the *crz1*Δ mutant, indicating that the genes are upregulated in response to thermal stress. The two *S*. *cerevisiae* orthologs (*PMC1* and *CHS1*) are similarly upregulated in response to Ca^2+^ stress. To determine the significance of this overlap between the two gene sets, we performed a resampling test to evaluate the probability that the overlap occurred by chance (as described in Materials and Methods). The estimated probability for the *C*. *neoformans* and *S*. *cerevisiae* gene sets was 0.250, indicating that two-gene overlap between the two gene sets is likely due to chance, not conservation of a regulated gene.

In the pathogenic fungus *A*. *fumigatus*, 141 calcineurin-dependent genes were identified upon high Ca^2+^ stress [23]. From these we excluded four hypothetical proteins and found that from the remaining 137 there were 78 genes that have an ortholog in *C*. *neoformans*. When we compared the 102 genes identified from our study to the *A*. *fumigatus* gene set, we found that 56 genes have an ortholog in *A*. *fumigatus*, and from this subset, five genes (CNAG_00025 *VCX1*, CNAG_01232 *PMC1*, CNAG_02217 *CHS7*, CNAG_03412 *CTS1*, and CNAG_04737) have six orthologs that were regulated by the calcineurin pathway in *A*. *fumigatus* (**Fig 4B** and **S5 Table**). The five genes are downregulated in the *crz1*Δ mutant, indicating that the genes are upregulated in response to thermal stress. Of the five genes, two genes (CNAG_04737 and *CTS1*) have *A*. *fumigatus* orthologs that were differentially regulated. In the *A*. *fumigatus crzA*Δ mutant, *chiB1* (Afu7g08490) and Afu6g03450 are upregulated under Ca^2+^ stress. We repeated the resampling test, and found that the estimated probability for the overlap between the *C*. *neoformans* and *A*. *fumigatus* gene sets was 0.002. The low probability of an overlap of at least six genes by chance suggests some conservation of calcineurin/Crz1 regulation between *C*. *neoformans* and *A*. *fumigatus*.

We further applied this analysis to compare the *S*. *cerevisiae* gene set with the gene set from *A*. *fumigatus*. From the OrthoMCL analysis, we found that 116 out of 144 *S*. *cerevisiae* calcineurin-dependent genes have orthologs in *A*. *fumigatus*, and 63 out of 137 *A*. *fumigatus* calcineurin-dependent genes have orthologs in *S*. *cerevisiae*. When we compared the two subsets, we found three *S*. *cerevisiae* genes (YGL006W *PMC1*, YLR350W *ORM2*, and YNL192W *CHS1*) overlapping with four *A*. *fumigatus* orthologs (**S5 Table**). The resampling analysis found a probability of 0.268 suggesting that this number of overlapping genes could occur by chance, as noted in the comparison between *C*. *neoformans* and *S*. *cerevisiae*.

This analysis shows that the genes regulated by the calcineurin-Crz1 pathway differ significantly between *C*. *neoformans* and *S*. *cerevisiae*, *C*. *neoformans* and *A*. *fumigatus*, and *A*. *fumigatus* and *S*. *cerevisiae*. Given that the gene sets of *S*. *cerevisiae* and *A*. *fumigatus* were obtained using different stress-inducing conditions, our results could suggest that under thermal stress, the *C*. *neoformans* calcineurin-Crz1 pathway may regulate a subset of genes that is different from the genes it may regulate under ionic stresses. But given that the genes regulated in *S*. *cerevisiae* and *A*. *fumigatus* differ significantly in response to the same stress condition (calcium ion stress) lends support to the conclusion that the output of the pathway differs between these species.

We found that the *C*. *neoformans* calcineurin-Crz1 pathway positively regulated both *PMC1* and the calcium ion transporter gene *VCX1*, unlike in *S*. *cerevisiae*, where the Vcx1 protein is negatively regulated by calcineurin post-transcriptionally and independently of Crz1 [12, 34–36]. In addition to *PMC1*, a small number of genes have been previously identified as calcineurin-dependent gene targets in *S*. *cerevisiae*: *FKS2*, which encodes β-1,3-glucan synthase; the P-type ATPases *PMR1* and *ENA1*; and the calcineurin regulator, *RCN1* [34, 36–38]. We found that *FKS1*, encoding the sole β-1,3-glucan synthase, and *MPK1*, encoding the downstream protein kinase in the PKC pathway, were regulated by Crz1 independently of calcineurin under thermal stress. Adler *et al*. previously concluded that Pkc1 positively regulates Crz1 under glucose starvation, through phosphorylation. From our RNA-sequencing analysis results, we propose that Crz1 is regulated by calcineurin under thermal stress, and that the PKC pathway may act antagonistically to calcineurin.

### Motif enrichment analyses

**Fig 5:**
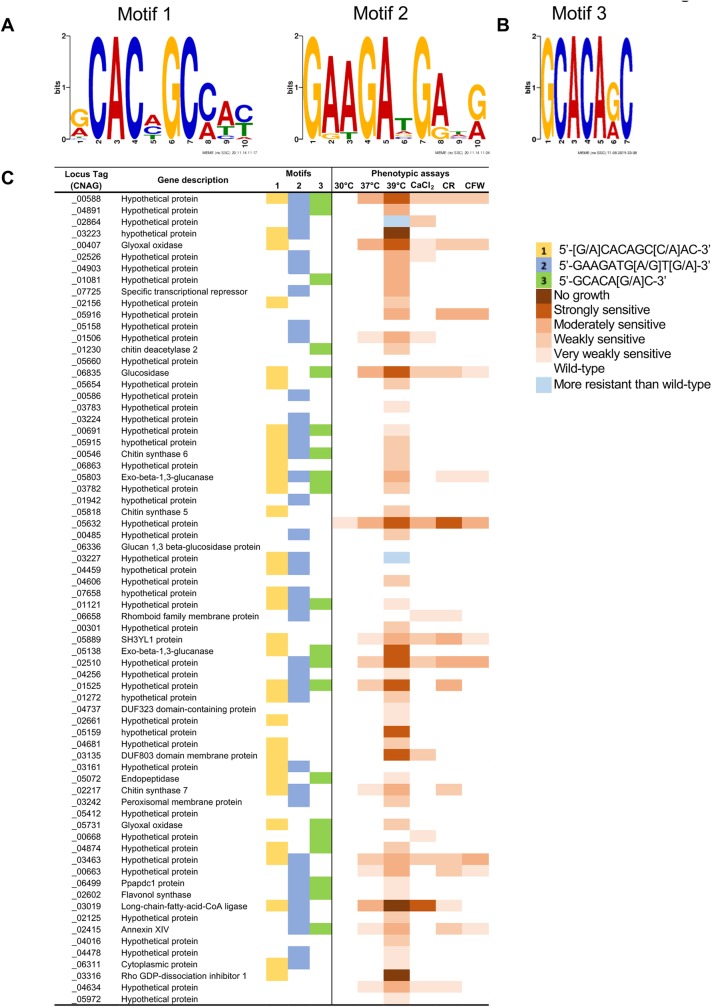
Genes regulated by the calcineurin-Crz1 pathway feature three motifs. **(A)** DNA motifs generated by MEME from promoter analysis of the 102 target genes. **(B)** DNA motif generated by DREME from promoter analysis of *CHS6* (CNAG_00546), and three other genes (CNAG_00588, CNAG_04891, and CNAG_00407). **(C)** Comparison of the presence or absence of the motifs against the phenotypic analyses of the target gene deletion mutants. CaCl_2_ = 0.5 M calcium chloride; CR = 1% Congo red; CFW = 5 mg/mL calcofluor white.

Crz1 target genes have been shown to contain a Crz1 binding motif in their promoter regions, also known as the calcineurin-dependent response element (CDRE). To identify the CDRE motif in *C*. *neoformans*, we performed a motif search employing MEME (Multiple Em for Motif Elucidation) with the *S*. *cerevisiae*, *C*. *albicans*, *M*. *oryzae*, and *A*. *fumigatus* CDRE motif sequences serving as consensus sequences [12, 22, 23]. However, we were unable to identify any unique motif sequences. We next attempted to identify the putative CDRE motifs with MEME by using 1 kb of upstream promoter sequences of the 102 calcineurin-Crz1 regulated genes and restricting the maximum size of the motif to 10 bp. Two putative motifs were identified: motif 1 (5’-[G/A]CACAGC[C/A]AC-3’) was found in 48 genes, and motif 2 (5’-GAAGATG[A/G]T[G/A]-3’) was present in 52 genes (**Fig 5A** and **S6 Table**).

A second attempt was performed using the motif discovery algorithm DREME (Discriminative Regular Expression Motif Elicitation), using 1 kb of the upstream promoter sequences of the *CHS6* gene (CNAG_00546), which has been found to be a target of Crz1, and three other genes (CNAG_00588, CNAG_04891, and CNAG_00407) that displayed the highest fold-change in expression levels in the RNA-seq analysis. Corresponding promoter sequences from the orthologs in *C*. *deneoformans*, *C*. *gattii*, and *C*. *amylolentus* were also used in order to increase specificity of the motif search. From this search, a third motif that is 7 bp in length (5’-GCACA[G/A]C-3’) was identified with an e-value of 1.3e^-4^ (**Fig 5B**). This motif is a shorter variant of motif 1 identified in the MEME analysis. We used Analysis of Motif Enrichment (AME) to check for motif enrichment among the promoter regions of the 102 calcineurin-Crz1 regulated genes, and found that the motif was enriched (*p*-value = 1.95e^-4^), and is present in the promoter regions of 29 genes. We found that 21 genes did not contain any of the three putative motifs, suggesting that these genes may be indirect targets of the calcineurin-Crz1 pathway (**S6 Table**).

**Fig 6:**
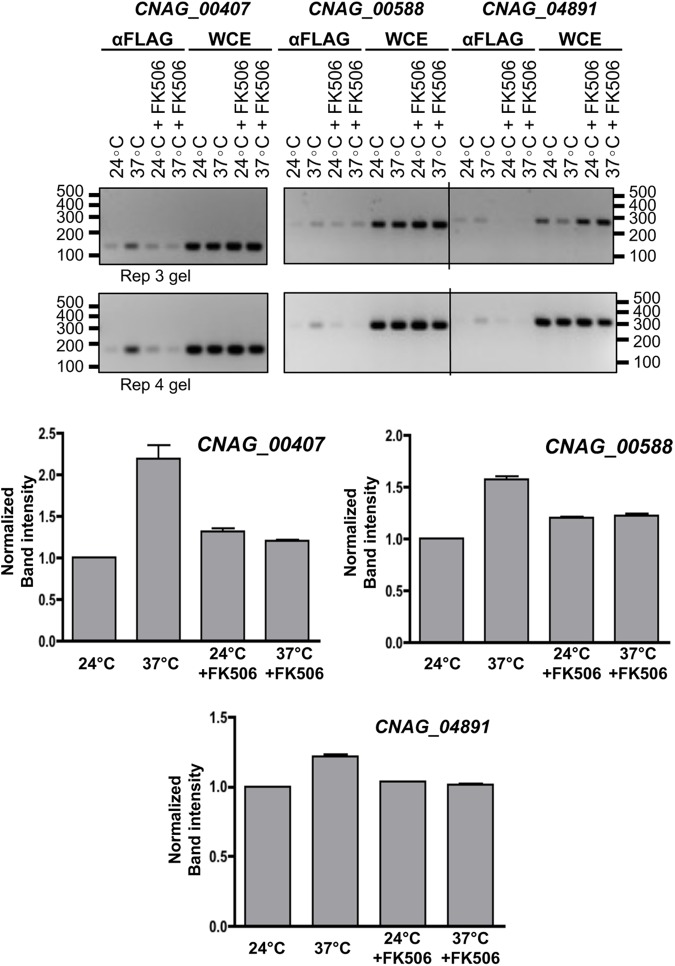
Crz1 binding of promoter regions increases under thermal stress. Cultures of the Crz1-4xFLAG strain were grown to exponential phase at 24°C, shifted to 37°C with and without FK506 for 30 min, and crosslinked with formaldehyde. Whole cell lysates (WCE) were prepared and ChIP-PCR was performed employing anti-FLAG nickel beads and specific DNA primers for three genes (CNAG_00588, CNAG_00407, and CNAG_04861) as described in material and methods. PCR DNA products for the DNA obtained from the anti-FLAG CHIP-PCR or from WCE-PCR were fractionated in agarose gels. Primers for each gene tested were designed to produce a product 150–280 bp long. Quantification of band intensity was analyzed using ImageJ, and values were normalized to the 24°C control. Results shown are representative of two biological replicates (labelled as Rep 3 gel and Rep 4 gel above).

To verify if genes containing the motifs were direct targets of Crz1, we performed ChIP-PCR using the *crz1*Δ mutant complemented with the Crz1-4xFLAG construct described in [31]. Cultures were grown at 24°C and then shifted to 37°C for 1 hour, with and without FK506, with biological duplicates. Three genes with the highest fold-changes (CNAG_00588, CNAG_00407, CNAG_04819) were selected, and primers were designed to amplify 150–280 bp segments of the promoter regions containing the motifs (**S4 Fig**). We observed that in general, Crz1 binding intensity was increased upon temperature shift to 37°C compared to 24°C and this increase was inhibited by exposure to FK506 (**Fig 6**).

### Deletion mutants of genes regulated by the calcineurin-Crz1 pathway display differential phenotypes

Of the 102 genes regulated in a calcineurin-Crz1 dependent manner, we were able to phenotypically assay 70 gene deletion mutants from the *C*. *neoformans* gene deletion collection deposited at the FGSC by the Madhani lab [39]. From the group of 70 mutants tested, 10 mutants displayed similar phenotypes to wild-type when assayed on media containing 0.5 M CaCl_2_, 1% Congo red, and 5 mg/mL calcofluor white, or growth at 37°C and 39°C. 14 mutants presented with an intermediate phenotype compared to the *cna1*Δ and *crz1*Δ mutants at 37°C, while 55 mutants presented with similar temperature sensitive growth phenotypes to the *crz1*Δ mutant (**Fig 5C, S5** and **S6 Figs**). One mutant (*cnag_03463*Δ) encoding a protein of unknown function displayed slower growth across all conditions assayed (**S5 Fig**). Interestingly, two deletion mutants (*cnag_02864*Δ and *cnag_03227*Δ) displayed a slightly increased resistance at 39°C (**S6 Fig**). In summary, 60 out of 70 genes regulated by the calcineurin-Crz1 pathway, when mutated, conferred stress sensitivity to different degrees. This represents a significant enrichment of this phenotype, reinforcing the model that genes regulated by the calcineurin-Crz1 pathway have roles in stress resistance.

## Discussion

The transcription factor Crz1 has been shown to be a major downstream effector of calcineurin in various model and pathogenic fungi. Recent studies have identified CNAG_00156 as the putative *C*. *neoformans CRZ1* ortholog, with differing conclusions [28–30]. We identified Crz1 from a calcineurin-dependent phosphoproteomic screen in a recent study [31]. Reciprocal protein BLAST searches suggest that CNAG_00156 is an ortholog of *S*. *cerevisiae* Crz1, and similar results were obtained with Crz1 from *C*. *albicans* and *A*. *fumigatus* CrzA. At room temperature, we observed that wild-type Crz1 is distributed throughout the cytosol. At 37°C, Crz1 translocated into the nucleus in a calcineurin-dependent manner; inhibition of calcineurin at 37°C by the presence of FK506 prevented the translocation of Crz1 into the nucleus. We identified two putative calcineurin docking domains (i) ^451^PMICIQ^456^ and (ii) ^868^PALSIS^873^. Deletion of the ^451^PMICIQ^456^ domain prevented the translocation of Crz1 into the nucleus under thermal stress (**Fig 1C**), suggesting that this domain is important for calcineurin interaction with Crz1, and its dephosphorylation activity. Lev *et al*. had previously predicted three candidates: (i) ^659^PRLDPD^664^, (ii) ^394^PNIVTQ^399^, and (iii) ^451^PMICIQ^456^, and mutagenized the latter two motifs to PNIddQ and PMIddQ respectively, and did not observe any effects on nuclear targeting of mutant proteins [29]. In *S*. *cerevisiae*, the calcineurin docking domain PIISIQ in Crz1 has been demonstrated to mediate direct binding with calcineurin, and loss of the motif compromises regulation by calcineurin and results in sustained cytosolic localization of Crz1 under stress conditions [32].

**Fig 7:**
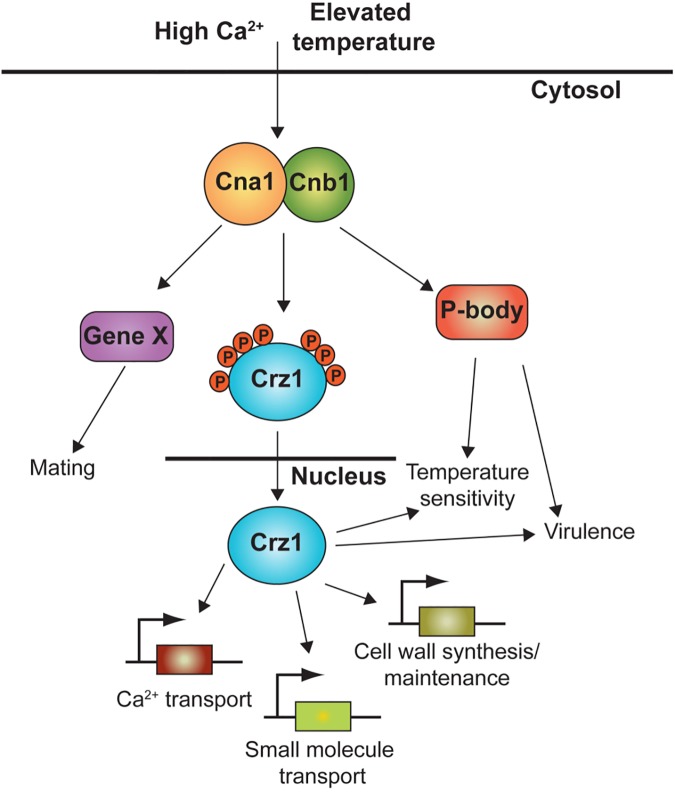
Model of calcineurin-Crz1 signaling pathway in *C*. *neoformans*. Under stress conditions such as high Ca^2+^ concentration or thermal stress, calcineurin is activated and dephosphorylates Crz1, resulting in its translocation to the nucleus. In the nucleus, Crz1 upregulates the expression of stress response genes, such as those involved in cell wall maintenance or remodeling and small molecule transport. Calcineurin also translocates to P-bodies and stress granules [27] where it may act upon other factors that influence mRNA expression or stability to operate a bifurcated transcriptional/post-transcriptional stress response network necessary for fungal virulence.

Concurrent with earlier reports, we found that the *crz1*Δ mutant was sensitive to cell wall perturbing agents (SDS and Congo red), and to high concentrations of CaCl_2_. Deletion of *CRZ1* did not compromise the ability to grow at the host physiological temperature (37°C), but we found that growth at higher temperatures (39°C) was inhibited (**Fig 2A**). Loss of *CRZ1* did not affect hyphal formation or sporulation in a bilateral genetic cross. We observed that the loss of *CRZ1* resulted in attenuated virulence in an animal infection model, albeit to a much lesser extent observed for the *cna1* calcineurin mutant, which is avirulent. This virulence defect was largely remediated to wild-type when the *crz1*Δ mutant was complemented with the wild-type *CRZ1* gene. These phenotypic results support roles for Crz1 in cell wall integrity and calcium sequestration in the calcineurin signaling pathway, and also indicate that calcineurin acts on other factors that function to govern thermotolerance and mating (**Fig 7**).

To identify the downstream target genes of the calcineurin-Crz1 signaling pathway, we employed RNA-sequencing on cultures grown at 24°C and 37°C. At the resting, non-induced state (24°C), we found that only a small number of genes were differentially expressed in the *cna1*Δ mutant, and of these genes, five were also differentially expressed in the *crz1*Δ mutant. However, under thermal stress, we found 495 genes were regulated by calcineurin and 161 genes by Crz1. Comparing the two gene datasets, we identified 102 genes as being differentially regulated in a calcineurin-Crz1-dependent manner; of these, 99 genes were down-regulated in both the *cna1*Δ and *crz1*Δ mutants, indicating that under thermal stress, the calcineurin-Crz1 pathway acts predominantly to positively regulate gene expression. Consistent with our hypothesis that Crz1 acts downstream of calcineurin to regulate cell wall integrity based on the phenotypic assays, 11% of the genes encode proteins that contribute to cell wall integrity/maintenance; examples of these include: *CHS7*, *CHS6*, *CHS5*, *KRE6*, *CDA2*, *CTS2*, *EXG1*, and *BGL2*. We also identified genes involved in membrane transport (*VCX1*, *PMC1*, and *RIM1*), signaling and transcription, and amino acid and carbohydrate metabolism.

Employing both MEME and DREME analyses, we identified three putative CDRE motifs: (1) 5’-[G/A]CACAGC[C/A]AC-3’, (2) 5’-GAAGATG[A/G]T[G/A]-3’, and (3) 5’-GCACA[G/A]C-3’ present within the promoter regions of 81 out of the 102 genes regulated by the calcineurin-Crz1 pathway. All three motifs bear little similarity to the CDRE motifs previously identified in the ascomycetes *S*. *cerevisiae*, *C*. *albicans*, and *A*. *fumigatus*. Interestingly, the three motifs were also present in the promoter regions of 41 out of the 59 genes that were regulated by Crz1 independently of calcineurin (**S7 Table**). Under different stress conditions, such as ionic stresses, these genes may be regulated in a calcineurin-dependent manner.

Apart from *PMC1*, which was identified among the genes regulated by the calcineurin-Crz1 pathway, we did not identify either *ENA1* or *PMR1* as being regulated by this pathway as has been shown in *S*. *cerevisiae* [12, 40], or by calcineurin independently of Crz1. This result is in accord with previous studies demonstrating that the loss of *CRZ1* did not result in Na^+^ sensitivity in *C*. *neoformans* [29]. In *S*. *cerevisiae*, the calcineurin-Crz1 signaling pathway is regulated by a feedback loop involving the transcriptional activation of *RCN1*. The *CBP1* gene (Calcineurin Binding Protein 1) encodes a homolog of *RCN1* in *C*. *neoformans* that has previously been shown to interact with the calcineurin A catalytic subunit and play a role in mating. Interestingly, Cbp1 was not regulated by either calcineurin or Crz1 under thermal stress in *C*. *neoformans*, based on our RNA-sequencing analysis. Moreover, *CBP1* is not needed for growth at high temperature and plays a modest role in virulence [41, 42]. Taken together, these results indicate that the thermally activated calcineurin-Crz1 pathway in *C*. *neoformans* differs from that responding to Ca^2+^ stress in *S*. *cerevisiae*, and suggests that either the calcineurin-Crz1 pathway varies in response to different stresses or the network has been rewired during fungal evolution.

Although the core signaling cascade involving calmodulin, calcineurin, and Crz1 is highly conserved across species, the downstream effectors seem to have diverged. Even within the ascomycete calcineurin-Crz1 signaling pathway, downstream target genes are different in response to different stimuli [14, 23]. Comparative analyses of the 102 *C*. *neoformans* calcineurin-Crz1 dependent genes against the gene sets previously identified for *S*. *cerevisiae* [11] and *A*. *fumigatus* [23] supports this hypothesis. Comparison between the *C*. *neoformans* and *S*. *cerevisiae* gene sets revealed that while 30 of the 102 genes have *S*. *cerevisiae* orthologs, only two of these genes are regulated by the calcineurin-Crz1 pathway in both species, and the probability of the observed overlap (0.250) suggest that the occurrence is most parsimoniously by chance. When the same analysis was performed comparing the *C*. *neoformans* and *A*. *fumigatus* gene sets, we found that 56 of the 102 *C*. *neoformans* genes have orthologs in *A*. *fumigatus*. From these 56 orthologs, five genes are calcineurin-Crz1 targets in both fungi, with a lower probability of 0.002, suggesting some modest conservation in the downstream target genes between the two fungal species. While relatively few orthologs are commonly regulated in all three species, some of the remaining downstream target genes may play related functions, such as in cell wall integrity/maintenance, ionic stress resistance, and vacuolar calcium sequestration. This plasticity may reflect network rewiring during evolution, allowing each species to cope with the stresses encountered in their particular environmental niches.

Additionally, Crz1 may not be the only transcription factor in *C*. *neoformans* acted upon by calcineurin. This is evident based on: 1) the intermediate phenotypes observed in the *crz1*Δ mutant at 37°C compared to the wild-type and the *cna1*Δ mutant, 2) attenuated virulence of the *crz1*Δ mutant, rather than the complete loss of virulence observed in the *cna1*Δ mutant, and 3) the regulation of other transcription factors by calcineurin, independently of Crz1, based on RNA-sequencing. Thus if rewiring occurred it could have happened by a mechanism similar to that proposed by Baker et al. involving the integration of a new transcriptional regulator into the existing network, as in the MAT**a** specific gene network rewiring in the Saccharomycota [43].

In summary, we have identified a suite of genes that is regulated by the calcineurin-Crz1 signaling pathway under thermal stress. Given the intermediate *crz1*Δ mutant phenotype, evidence for a calcineurin regulatory network impacting mRNA independently of Crz1, and the finding that calcineurin relocalizes to P-bodies and stress granules during thermal stress [27], calcineurin may act on factors in addition to Crz1 that govern mRNA expression or stability to operate a bifurcated transcriptional/post-transcriptional stress response network necessary for fungal virulence (**Fig 7**). Taken together, our findings suggest that the calcineurin-Crz1 stress response cascade has been maintained from ascomycetous to basidiomycetous fungi, but its output in *C*. *neoformans* seems to have been rewired during evolution. Future studies to further characterize the calcineurin-Crz1-regulated genes will likely illuminate links between the three major stress response pathways in *C*. *neoformans*, namely the alkaline stress response, HOG, and PKC pathways, and thereby provide new avenues for research into the identification of antifungal targets.

## Materials and methods

### Strains and plasmids

Fungal strains and plasmids constructed in this study are listed in **S8 Table**. All fungal strains listed in **S8 Table** were derived from *C*. *neoformans* var. *grubii* laboratory reference strain H99 (#4413), unless otherwise stated. Fungal transformation was carried out using the biolistic method [44]. Fungal strains were maintained on YPD agar (1% yeast extract, 2% bactopeptone, and 2% dextrose) supplemented with the relevant antibiotics and grown at 30°C.

The *crz1*Δ::*NAT* deletion mutant was generated using overlap PCR and biolistic transformation. To construct the Crz1-mCherry epitope-tagged strains for the site-directed mutagenesis, the Crz1 ORF was PCR amplified and fused with the mCherry fusion protein by splice overlap PCR, cloned into pCR2.1-TOPO (Invitrogen) to yield plasmid pXW15 which was verified by sequencing. The 7.96 kb BamHI Crz1-mCherry fusion fragment was subcloned into the BamHI site of the safe haven plasmid pSDMA25 [45], to generate plasmid pEC13 which was verified by sequencing. The recombinant Crz1-mCherry plasmid was linearized using the restriction enzyme AscI, and the *crz1*Δ::*NAT* deletion mutant was biolistically transformed. Transformants were screened for proper integration into the safe haven locus as previously described [45]. Targeted integration of each construct was confirmed by PCR amplification (5’ junction: JOHE40956/JOHE40957; 3’ junction: JOHE40958/JOHE41562). Tandem array integration was ruled out by PCR using inverse primers (JOHE41450/JOHE41451) that would only yield a product if two or more copies of the construct were tandemly integrated at the safe haven site. The *NAT* selectable marker in pSL04, which contains the nucleolar fluorescence marker GFP-Nop1, was replaced with the *HYG* selectable marker from pJAF15. The GFP-Nop1 construct was introduced into the recombinant Crz1-mCherry mutant strains by ectopic integration. All primers that were used in this study are listed in **S9 Table**. The Crz1^PMICIQ^Δ –mCherry plasmid was constructed by Gibson cloning, using the plasmid pEC13 as the template.

### Phenotypic assays

Strains were grown in liquid YPD media at 30°C overnight with shaking, and washed with PBS. Five 10-fold serial dilutions of each strain were made, and spotted out onto solid media. Unless otherwise stated, strains were incubated for 2 days at 30°C. YPD was supplemented with 2.5 M CaCl_2_ solution, mixed to achieve a final concentration of 0.35 M, 0.4 M, or 0.5 M of CaCl_2_. YPD was supplemented with 20% SDS, mixed to achieve a final concentration of 0.02% or 0.03% SDS. Congo red was added to YPD to a final concentration of 1% (w/v). Calcofluor white was added to final concentrations of 4 mg/mL or 5 mg/mL. MS mating medium was prepared as previously described [25]. Strains of opposite mating types were grown in liquid YPD media at 30°C overnight with shaking, and washed with PBS. Equal volumes of cell suspensions of each mating type were mixed, spotted onto MS mating media, and incubated at room temperature in the dark for 12 days before visualization using a Nikon Eclipse E400 microscope with an attached DXM200F digital camera.

### Microscopy

Strains were grown overnight in liquid YPD medium at room temperature with shaking. Cells were then diluted to OD_600_ = 0.1–0.2 with fresh YPD medium (20 mL), and grown for a further five hours, until OD_600_ = 0.5–0.6 was achieved. A 5 mL aliquot of the cell culture was incubated in a 37°C water bath for 15 min. Cells incubated at 24°C and at 37°C were then fixed with 4% formaldehyde for 15 min, and washed with KPO_4_/sorbitol buffer. To verify that translocation of Crz1 is due to calcineurin activity, FK506 (final concentration = 1 μg/mL) was added to the cultures, and cells were incubated for a further 15 min before being shifted to 37°C. To observe the localization of the Crz1^PMICIQ^Δ construct, cells were shifted to 38°C for 20 min prior to formaldehyde fixing. Cell suspensions were spotted onto slides with a layer of 1.5% YNB-agarose and covered with a coverslip. Cells were visualized by direct fluorescence microscopy using a Zeiss Axioskop 2 Plus microscope and AxioVision 4.6 image acquisition software. Nuclear fluorescence was quantified by ImageJ software.

### RNA isolation and real-time PCR

Strains were grown overnight at 24°C with shaking, until an OD_600_ = 0.5 was reached. Cell cultures were divided in two. One half was incubated at 24°C while the other half was transferred to 37°C, for a further 2 hours. Cells were collected by centrifugation, frozen at -80°C, and then lyophilized overnight. Total RNA was isolated from lyophilized cells using Trizol solution (Invitrogen Life Technologies, Carlsbad, CA) and the Qiagen RNeasy Mini kit (Qiagen, Valencia, CA). All RNA samples were collected in biological triplicates.

For quantitative PCR, total RNA was reversed transcribed into first strand cDNA by oligo dT priming using the AffinityScript cDNA synthesis kit according to the manufacturer’s instructions (Ailgent Technologies, Santa Clara, CA). The resulting cDNA was diluted with ultra-pure water. Real-time PCR was performed using the Brilliant III Ultra-Fast SYBR Green QPCR master mix (Agilent Technologies) and the Applied Sciences StepOnePlus Real Time-PCR system. Primers are listed in **S9 Table**.

### RNA-sequencing data analysis

Direct polyA RNA sequencing was performed by the High Throughput Sequencing Facility at the University of North Carolina, Chapel Hill. Preliminary quality analysis of the raw FASTQ files was conducted using FastQC [46], quality filtering was then performed using fastq-mcf [47] to remove sequencing adapters (adapter sequences supplied by Illumina, Inc) and trim low quality bases. Tophat2 [48] was used to map the processed reads to the H99 genome and transcriptome as determined from the genome annotation (version 2, downloaded from the Broad Institute) [49], using the following parameters: "—transcriptome-max-hits 1—max-multihits 1—max-intron-length 4000—library-type fr-unstranded—no-coverage-search". The program htseq-count [50] was used to determine read counts per gene from the BAM file output by Tophat; the htseq-count parameters used were "—stranded = no—type = exon—idattr = gene_id". A custom script was developed in the R programming language [51] to analyse the count data in order to do quality testing, identify differentially expressed genes, and generate figures. This script used the R packages DESeq2 [52], gplots [53], RColorBrewer [54], and optparse [55].

Preliminary data analysis led us to excluded from analysis one of the *cna1*Δ 37°C replicates, which was found to contain reads mapping to the deleted portion of CNA1. This preliminary analysis also indicated that the *CRZ1* complemented *crz1*Δ strains have much higher expression of CRZ1 than the wild-type strain.

The RNA-Seq data discussed in this publication have been deposited in NCBI's Gene Expression Omnibus (GEO) [56] and are accessible through GEO Series accession number GSE93005 (https://www.ncbi.nlm.nih.gov/geo/query/acc.cgi?acc=GSE93005). The custom programs developed for processing and analyzing the RNA-Seq data are available in a GitHub software repository (https://github.com/granek/crne_cna1crz1_rnaseq). This repository includes all programs, support files, and instructions for automatically replicating all analyses presented here using the data available from GEO.

### Orthology search and resampling test analysis

Orthologs were as determined by FungiDB [57], using OrthoMCL. The list of genes regulated by the calcineurin-Crz1 pathway in *S*. *cerevisiae* was taken from [11]; the study identified 163 regulated genes, of which 153 genes are regulated in response to Ca^2+^ stress. Nine genes (YNL043C, YMR007W, YPR197C, YMR304C-A, YDL011C, YDL172C, YPR170C, YDR535C, YGL165C) listed as “hypothetical genes” [11] were omitted from consideration, as the gene annotations were not found in FungiDB, to result in 144 genes that were further analyzed. The list of genes regulated by the calcineurin-Crz1 pathway in *A*. *fumigatus* was taken from [23]; 4 genes (Afu6g12810, Afu6g11860, Afu1g00190, Afu2g07390) listed as “hypothetical genes” were omitted from consideration as the gene annotations were not found in FungiDB. Further, FungiDB gives Afu2g05330 as the current name for the gene listed as Afu2g05325 in [23], so Afu2g05330 was used in this analysis. In total, 144 genes from *S*. *cerevisiae*, 137 genes from *A*. *fumigatus* and 102 genes from *C*. *neoformans* were analyzed.

The resampling test was performed to examine the likelihood that the overlap in regulated gene sets were to occur by chance. Briefly, sets of genes equivalent in size to the number of regulated genes with orthologs were randomly selected: 30 from all *C*. *neoformans* genes with an ortholog in *S*. *cerevisiae*, and 79 from all *S*. *cerevisiae* genes with an ortholog in *C*. *neoformans*. These two sets were compared to determine the number of orthologs shared between these two sets. This resampling procedure was repeated 1000 times, and the proportion of times that a simulated overlap was equal to or larger than the true overlap was tallied to give a probability estimate. The same test was carried out between *C*. *neoformans* (56 genes) and *A*. *fumigatus* (78 genes), as well as *S*. *cerevisiae* (116 genes) and *A*. *fumigatus* (63 genes). Scripts and data for replicating the resampling analysis are available in the GitHub software repository (https://github.com/granek/crne_cna1crz1_rnaseq).

### Motif discovery

To perform motif discovery, we employed the CDRE fungal motif sequences as indicated in the results section and the MEME suite was employed through use of the MEME webserver. In order to identify candidate motifs, both MEME [58] and DREME [59] were employed, with the input sequences randomly shuffled to provide a negative data set for DREME. Candidate motifs were tested for enrichment in the upstream 1 kb promoter sequences of candidate calcineurin-Crz1 regulated genes using AME [60].

### Chromatin immunoprecipitation

The ChiP analysis was conducted as previously described with some modifications [61]. Wild-type and *crz1*Δ + Crz1-4xFLAG strains were grown in liquid YPD media overnight at 30°C with shaking. Overnight cultures were diluted to an OD_600_ = 0.2–0.3 with fresh media, and further incubated at room temperature for 4 h. FK506 (final concentration 10 mM) was then added to two sets of cultures; one set of cultures was incubated at 37°C and the other at room temperature for a further 1 h. The same was performed for two sets of cultures without FK506. Formaldehyde (1% final concentration) was added to each culture, and incubated at room temperature for 20 min with shaking. Glycine (final concentration 125 mM) was added to quench the reaction, with 5 min incubation at room temperature with shaking. Fixed cells were pelleted and washed with PBS + 125 mM glycine twice. Cells were suspended with 1 mL ChIP lysis buffer (50 mM HEPES pH 7.4, 140 mM NaCl, 1% Triton X100, 1 mM EDTA), and 100 μL volume of acid washed glass beads were added. Cells were bead-beat three times for 1 min at 4°C with 1 min rest intervals and cell lysates were collected. Cell lysates were sonicated (12 cycles of 10 s pulses, followed with 5 min incubation on ice between each interval) and cleared by centrifugation. An aliquot of each lysate sample was kept as the lysate input controls. Lysates were immunoprecipitated using anti-FLAG nickel beads (Invitrogen/Life technologies) according to manufacturer’s protocol. Immunocomplexes were eluted by adding 250 μL of elution buffer (TE buffer, 1% SDS, 0.1M NaHCO_3_) to the beads, followed by a 5 min incubation at 65°C, prior to 15 min incubation at room temperature with constant rotation. The beads were then pelleted and the supernatant was collected. This step was repeated again to get a total volume of 500 μL. The eluates were de-crosslinked by adding 10 μL of 5M NaCl, and overnight incubation at 65°C. Following incubation, 10 μL of 0.5M EDTA, 20 μL of 1M Tris-HCl pH 6.8, and 2 μL of 20 mg/mL Proteinase K were added, and the eluates were further incubated for 1–2 hr at 45°C. Following extraction with phenol-chloroform-isoamyl alcohol, DNA was precipitated overnight at –20°C by adding 1 μL glycogen, 60 μL 3M NaOAc, and 1 mL 100% ethanol. DNA was pelleted by centrifugation at 13,000 rpm for 30 min at 4°C, and resolved in Ultra-Pure water and treated with 20 μg/mL RNaseA, before storage at -20°C.

DNA concentration was measured by Nanodrop, and diluted to an equimolar concentration for all samples. ChIP PCRs were done under typical laboratory PCR conditions with 1x ExTaq Buffer, 400nM dNTPs, 200nM forward primer, 200nM reverse primer, 0.625 units of ExTaq (Clonetech) and 1.2ul ChIP DNA template. Primers for each gene used can be found in **S9 Table**. PCR products were run on a 2% agarose gel containing 500μg/mL of ethidium bromide and imaged using the BioRad Gel Doc System.

### Virulence assays

*Galleria mellonella* were infected as previously described [62]. Cells were grown overnight in liquid YPD medium at 30°C with shaking, washed with PBS and suspended in the same buffer. Cell density was calculated using a hemocytometer and cell suspensions of 10^6^ cells/mL were prepared. Groups of 12 larvae were injected with 4 μL of the cell suspension (4 x 10^3^ cells) through the proleg; one group of larvae was injected with PBS as a control. The larvae were incubated at 37°C, and death was monitored daily for 15 days. Larvae that pupated during the 15-day period were censored. *G*. *mellonella* infections were performed in triplicate.

5–6 week old BALB/c female mice were used for the murine infection model. Groups of 10 mice were infected with wild-type, *crz1*Δ mutant, and *crz1*Δ *+ CRZ1* complemented strains (10^5^ cells) using the intranasal inhalation model previously described [63]. Mice were monitored and weighed daily; mice that appeared moribund or weighed less than 20% of their initial starting weight were euthanized.

Survival curves for both infection models were adjusted using the Kaplan-Meier method and estimation of differences in survival were analyzed using the log rank and Wilcoxon tests with GraphPad Prism software (GraphPad, San Diego, CA). A *p*-value below 0.05 was considered significant.

### Ethics statement

All experiments and animal care were conducted in accordance with the ethical guidelines of the Institutional Animal Care and Use Committee (IACUC) of Duke University Medical Center (DUMC). The DUMC IACUC approved all of the vertebrate studies under protocol number A245-13-09. Mice studies were conducted in the Division of Laboratory Animal Resources (DLAR) facilities that are accredited by the Association for Assessment and Accreditation of Laboratory Animal Care (AAALAC).

## Supporting information

S1 Fig Identification of a *bona fide* Crz1 ortholog in *C*. *neoformans*. **(A)** Reciprocal protein BLAST results of *C*. *neoformans* Crz1 (CNAG_00156) against *S*. *cerevisiae* Crz1; YNL027W, *C*. *albicans* Crz1; ORF19.7359, *C*. *glabrata* Crz1; CAGLOM06831g, *A*. *nidulans* crzA; AN5726, *A*. *fumigatus* crzA; Afulg06900, *S*. *pombe* Prz1; SPAC4G8.13c, and *M*. *oryzae* Crz1; MGG_05133. BLASTp analyses were performed using the FungiDB and BROAD portals. (**B)** Phylogenetic tree of fungal Crz1 proteins inferred using the Maximum Likelihood method using the Dayhoff matrix based model. Bootstrap values and branch lengths are indicated. *Cn*, *C*. *neoformans*; *Ca*, *C*. *amylolentus*; *Tw*, *T*. *wingfieldii*; *Mo*, *M*. *oryzae*; *Af*, *A*. *fumigatus*, *An*, *A*. *nidulans*; *Sc*, *S*. *cerevisiae*; *Cg*, *C*. *glabrata*; *Cal*, *C*. *albicans*; *Sp*, *S*. *pombe*. Schematic representations of the fungal Crz1 proteins with the PolyQ and zinc-finger domains, serine-rich regions and the PxIxIT domains indicated.
(TIF)

S2 Fig Calcineurin docking domain motif in *C*. *neoformans*. Two candidate motifs were identified in *C*. *neoformans*: ^868^PALSIS^873^ and ^451^PMICIQ^456^. The PALSIS motif is conserved between the different *Cryptococcus* species, while the PMICIQ motif is conserved in the pathogenic species complex. The green box represents the PolyQ domain; red boxes represent the zinc finger domains.
(TIF)

S3 Fig Real-time qPCR to validate differential gene expressions. Quantitative real-time qPCR was performed to validate the results obtained from RNA sequencing. Of the 22 genes analyzed, 20 genes showed very similar fold changes in expression to that detected by RNA-sequencing. CNAG_01525, CNAG_01230, CNAG_01081 and CNAG_06499 displayed higher magnitude of fold-change in *cna1*Δ (KK1) compared to *crz1*Δ (AFA 3–3) in the RT-qPCR. CNAG_00546, CNAG_07406 and CNAG_07725 showed lower magnitude of fold-change in the RT-qPCR.
(TIF)

S4 Fig ChIP PCR to quantify Crz1 binding to promoter regions of target genes. Schematic diagram indicating the location of ChIP-PCR primers in the promoter regions of the genes tested. Primers for each gene tested were designed to produce a product 150–280 bp long.
(TIF)

S5 Fig Phenotypic analysis of calcineurin-Crz1 regulated genes. Wild-type (KN99α), *cna1*Δ (KK1), and *crz1*Δ (AFA1-4) strains and gene deletion mutants from the Madhani deletion collection were grown in YPD media, washed, and resuspended in PBS. Five 10-fold serial dilutions of each strain were spotted on YPD solid media, with the various additives as listed and incubated at 30°C for 48 h, unless otherwise stated. Strains were incubated at 39°C for 72 h before imaging. CFW: calcofluor white.
(TIF)

S6 Fig Phenotypic analysis of calcineurin-Crz1 regulated genes. Wild-type (KN99α), *cna1*Δ (KK1), and *crz1*Δ (AFA1-4) strains and gene deletion mutants from the Madhani deletion collection were grown in YPD media, washed, and resuspended in PBS. Five 10-fold serial dilutions of each strain were spotted on YPD solid media, with the various additives as listed and incubated at 30°C for 48 h, unless otherwise stated. Strains were incubated at 39°C for 72 h before imaging. CFW: calcofluor white.
(TIF)

S1 Table Genes differentially expressed in *cna1*Δ and *crz1*Δ mutants under non-induced conditions. Gene fold-change values from the *cna1*Δ and the *crz1Δ* mutant were compared against wild-type separately, and genes were deemed differentially expressed if the fold-change was ≥2-fold. Within each comparison, genes were organized in ascending log2FC values. Gene names and descriptions listed were identified using the FungiDB search portal; gene orthology was determined using the GO function. Log2FC = Log2 Fold change
(DOCX)

S2 Table Genes regulated by calcineurin, independent of Crz1, under thermal stress. Gene fold-change values from the *cna1*Δ mutant were compared against wild-type and genes were deemed differentially expressed if the fold-change was ≥2-fold. Within each function classification, genes were organized in ascending log2FC values. Gene names and descriptions listed were identified using the FungiDB search portal; gene orthology was determined using the GO function. Log2FC = Log2 Fold change
(DOCX)

S3 Table Genes differentially regulated by Crz1 under thermal stress, in a calcineurin-independent manner. Gene fold-change values from the *crz1*Δ mutant were compared against wild-type and genes were deemed differentially expressed if the fold-change was ≥2-fold. Within each function classification, genes were organized in ascending log2FC values. Gene names and descriptions listed were identified using the FungiDB search portal; gene orthology was determined using the GO function. Log2FC = Log2 Fold change
(DOCX)

S4 Table 102 genes are differentially expressed in *cna1*Δ and *crz1*Δ mutants under thermal stress. Genes are deemed differentially expressed if the fold-change was ≥2-fold. Within each classification, genes were organized in ascending log2FC values. Gene names and descriptions listed were identified using the FungiDB search portal; gene orthology was determined using the GO function. Log2FC = Log2 Fold change
(DOCX)

S5 Table List of calcineurin-Crz1 regulated genes shared in common across species. Orthologs were determined from the FungiBD database using OrthoMCL. Note that Afu3g10690 (*pmcB*) and Afu7g01030 (*pmcC*) are both homologs of CNAG_01232 and YGL006W (*PMC1*); both genes were downregulated under Ca^2+^ stress in *A*. *fumigatus crzA*Δ mutant.
(DOCX)

S6 Table Motif discovery in promoter regions of genes regulated by the calcineurin-Crz1 pathway. Genes are arranged based on fold-change. Color shading indicates the presence of CDRE motifs; yellow = 5’-[G/A]CACAGC[C/A]AC-3’; blue = 5’-GAAGATG[A/G]T[G/A]-3’; green = 5’-GCACA[G/A]C-3’.
(XLSX)

S7 Table Motif discovery in promoter regions of genes regulated by the Crz1 independently of calcineurin. Genes are arranged based on fold-change. Color shading indicates the presence of CDRE motifs; yellow = 5’-[G/A]CACAGC[C/A]AC-3’; blue = 5’-GAAGATG[A/G]T[G/A]-3’; green = 5’-GCACA[G/A]C-3’.
(XLSX)

S8 Table List of fungal strains and plasmids used in the study. (DOCX)

S9 Table List of primers. (DOCX)
