# Supplementary figures and images for: Elucidation of the calcineurin-Crz1 stress response transcriptional network in the human fungal pathogen Cryptococcus neoformans
Source: PLoS Genet. 2017 Apr 4;13(4):e1006667. doi: 10.1371/journal.pgen.1006667 (PMC5380312; doi:10.1371/journal.pgen.1006667)

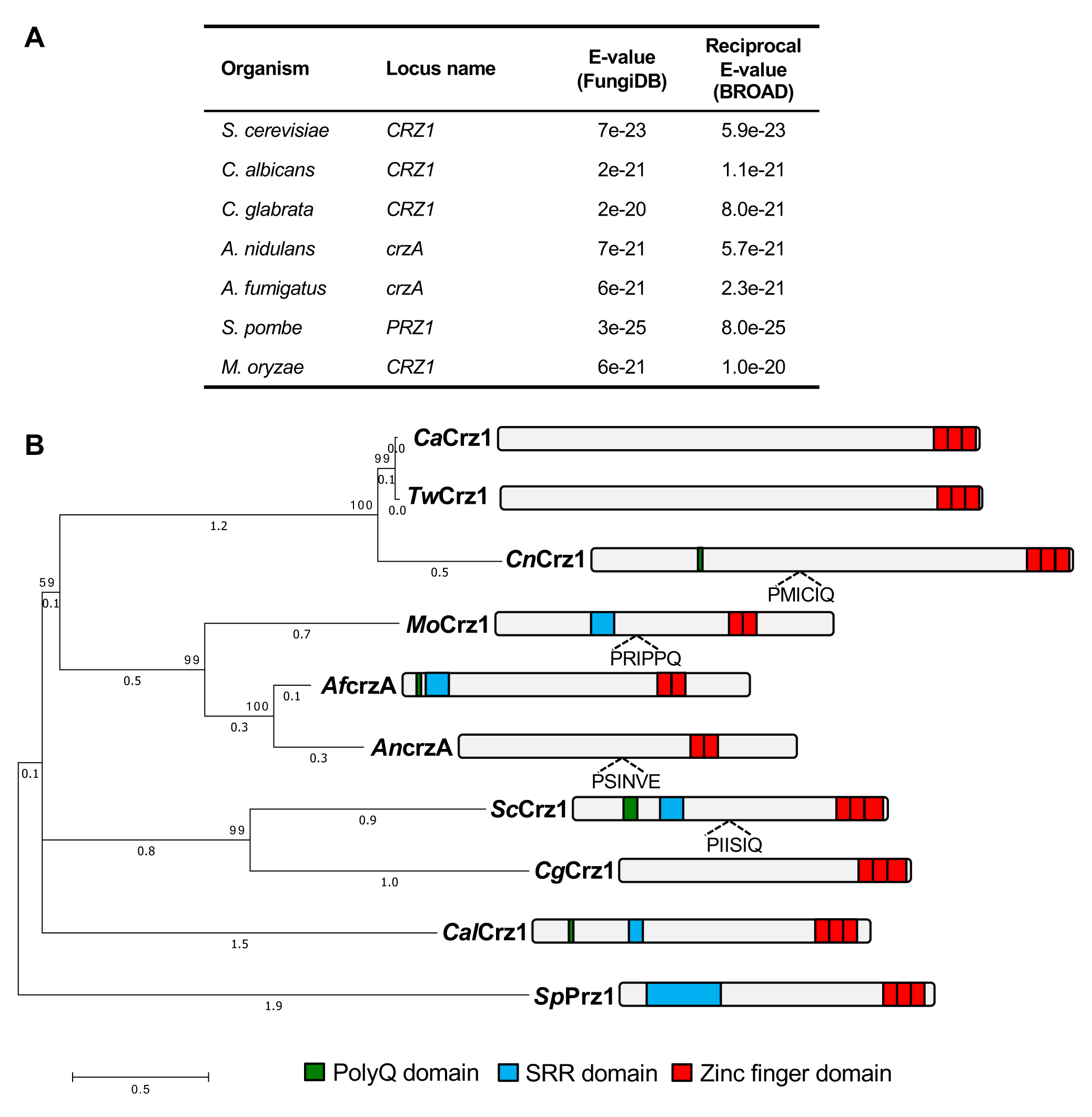

Supplement: S1 Fig — (A) Reciprocal protein BLAST results of C. neoformans Crz1 (CNAG_00156) against S. cerevisiae Crz1; YNL027W, C. albicans Crz1; ORF19.7359, C. glabrata Crz1; CAGLOM06831g, A. nidulans crzA; AN5726, A. fumigatus crzA; Afulg06900, S. pombe Prz1; SPAC4G8.13c, and M. oryzae Crz1; MGG_05133. BLASTp analyses were performed using the FungiDB and BROAD portals. (B) Phylogenetic tree of fungal Crz1 proteins inferred using the Maximum Likelihood method using the Dayhoff matrix based model. Bootstrap values and branch lengths are indicated. Cn, C. neoformans; Ca, C. amylolentus; Tw, T. wingfieldii; Mo, M. oryzae; Af, A. fumigatus, An, A. nidulans; Sc, S. cerevisiae; Cg, C. glabrata; Cal, C. albicans; Sp, S. pombe. Schematic representations of the fungal Crz1 proteins with the PolyQ and zinc-finger domains, serine-rich regions and the PxIxIT domains indicated. (TIF) [file pgen.1006667.s001.tif]

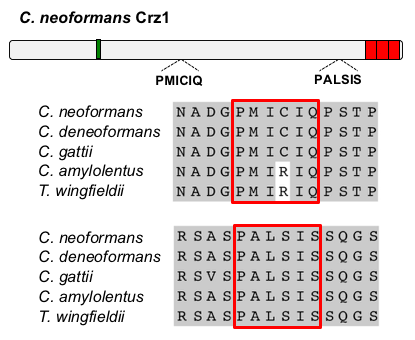

Supplement: S2 Fig — Two candidate motifs were identified in C. neoformans: 868PALSIS873 and 451PMICIQ456. The PALSIS motif is conserved between the different Cryptococcus species, while the PMICIQ motif is conserved in the pathogenic species complex. The green box represents the PolyQ domain; red boxes represent the zinc finger domains. (TIF) [file pgen.1006667.s002.tif]

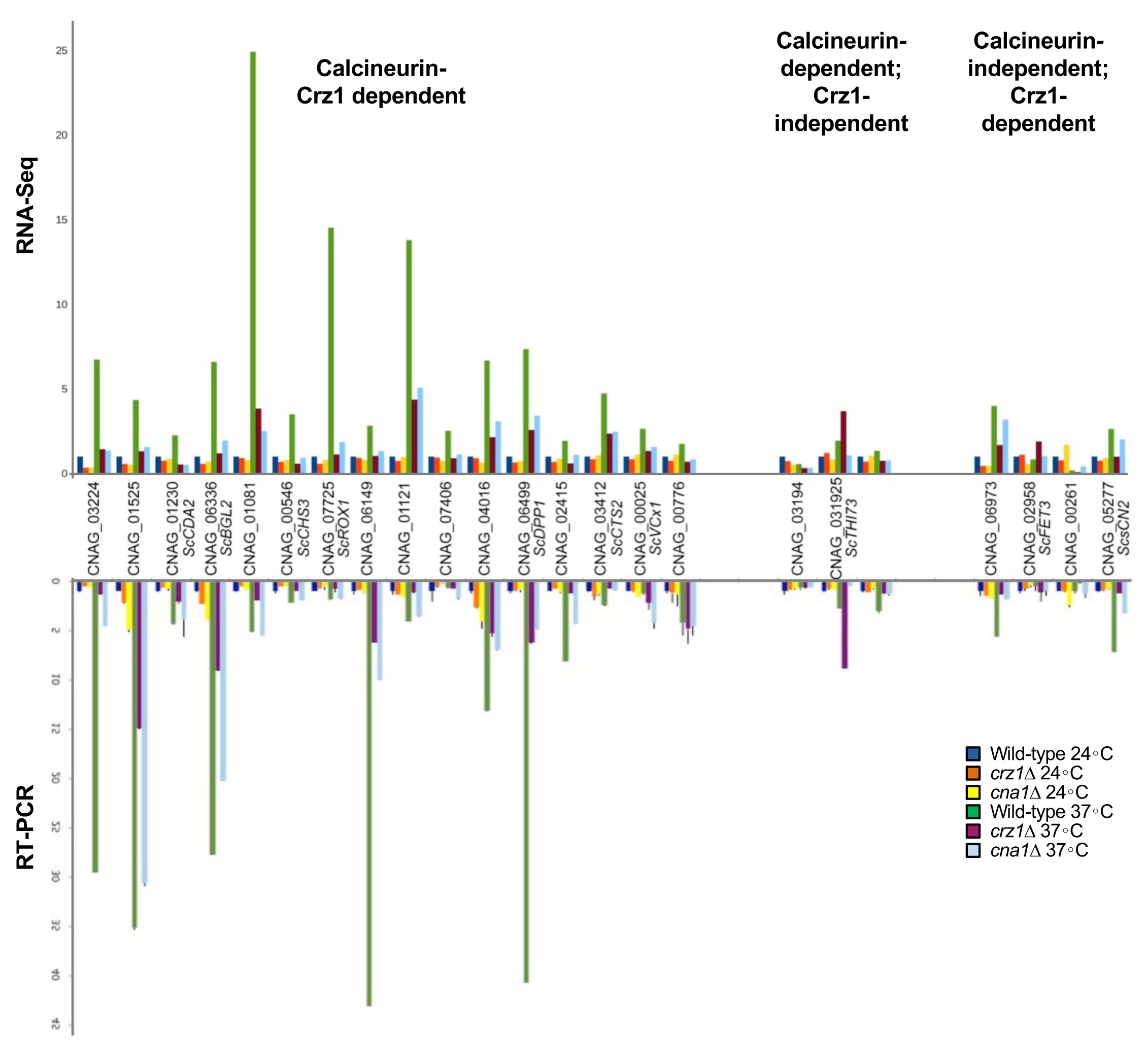

Supplement: S3 Fig — Quantitative real-time qPCR was performed to validate the results obtained from RNA sequencing. Of the 22 genes analyzed, 20 genes showed very similar fold changes in expression to that detected by RNA-sequencing. CNAG_01525, CNAG_01230, CNAG_01081 and CNAG_06499 displayed higher magnitude of fold-change in cna1Δ (KK1) compared to crz1Δ (AFA 3–3) in the RT-qPCR. CNAG_00546, CNAG_07406 and CNAG_07725 showed lower magnitude of fold-change in the RT-qPCR. (TIF) [file pgen.1006667.s003.tif]

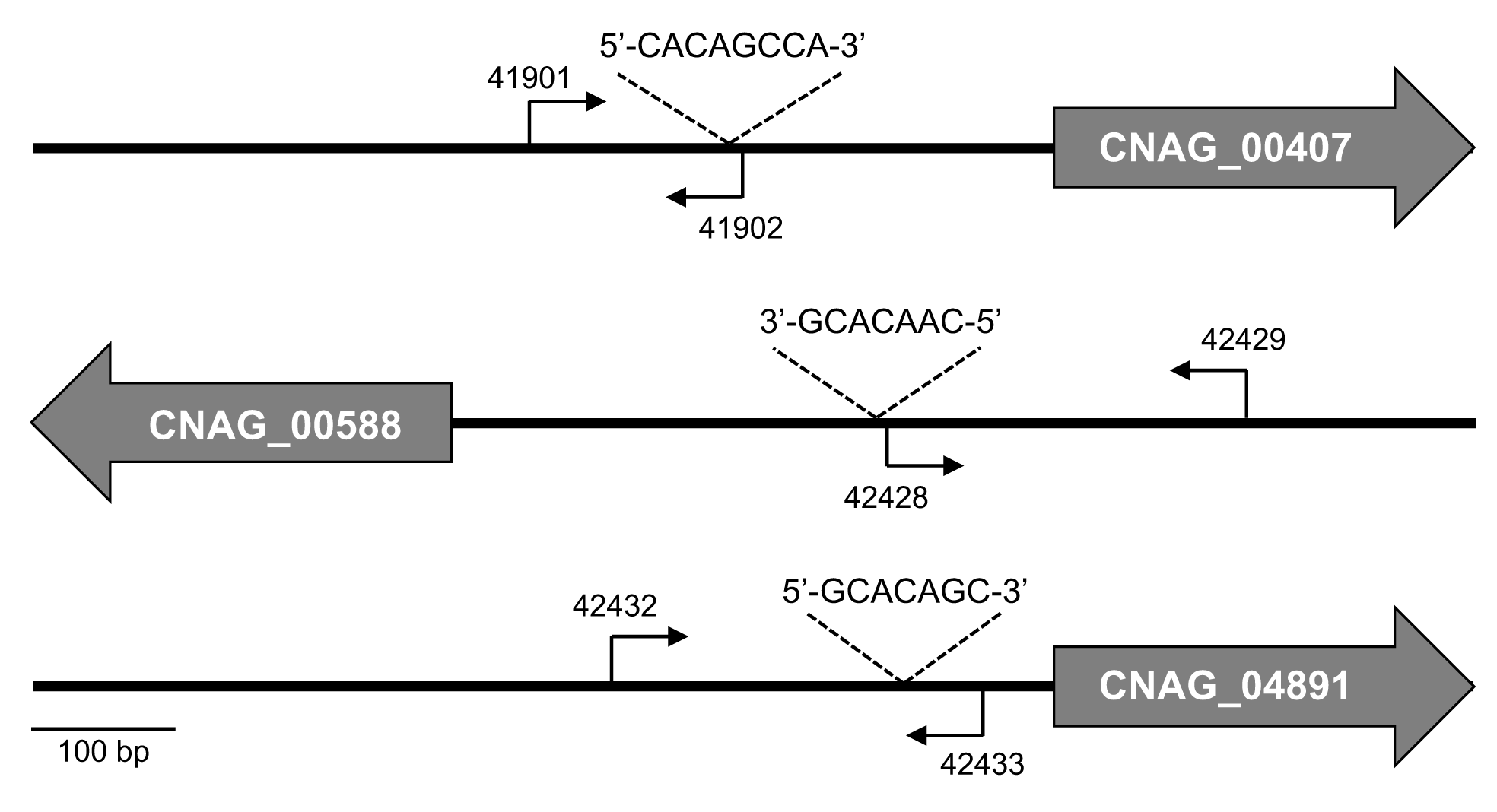

Supplement: S4 Fig — Schematic diagram indicating the location of ChIP-PCR primers in the promoter regions of the genes tested. Primers for each gene tested were designed to produce a product 150–280 bp long. (TIF) [file pgen.1006667.s004.tif]

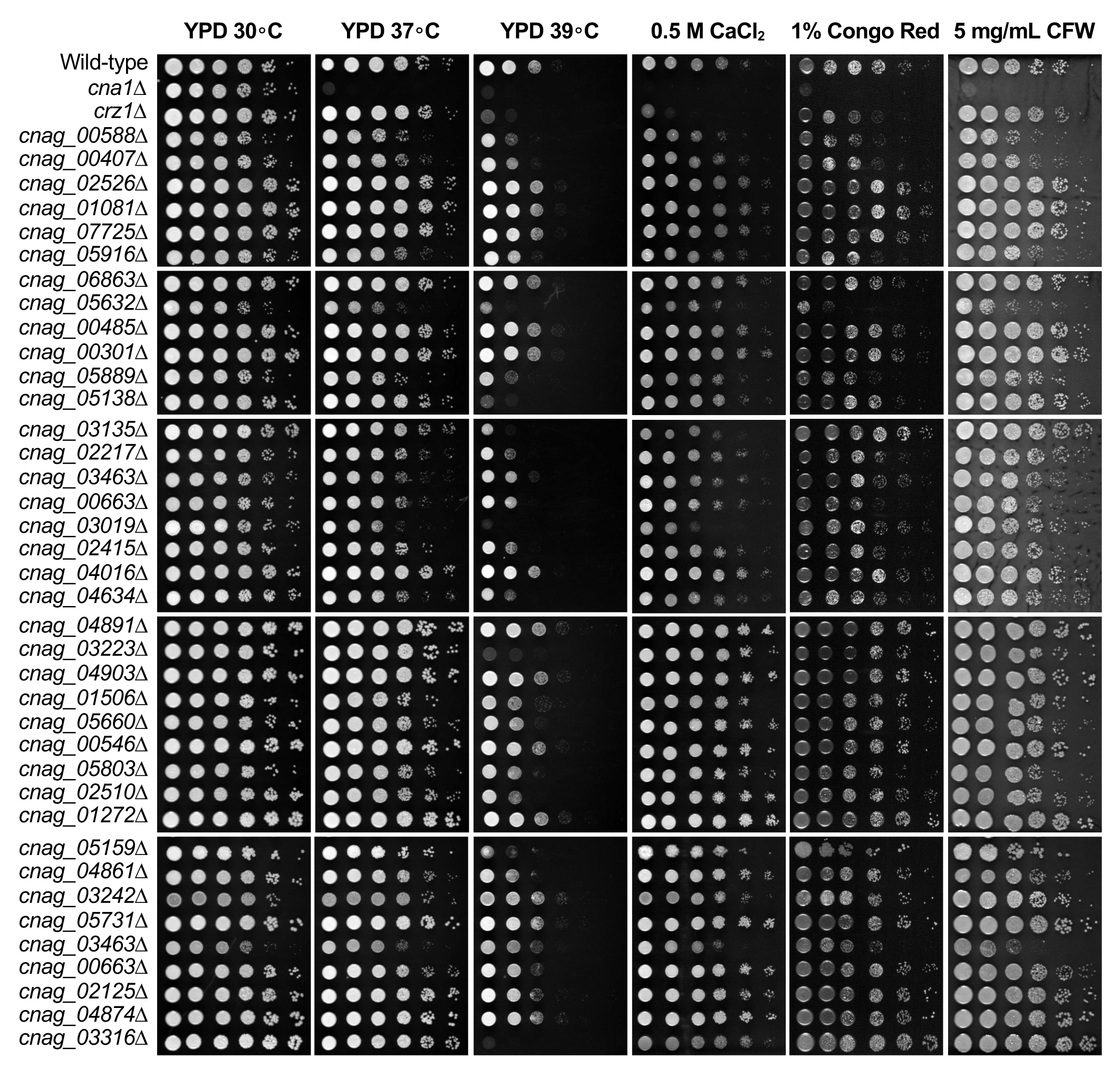

Supplement: S5 Fig — Wild-type (KN99α), cna1Δ (KK1), and crz1Δ (AFA1-4) strains and gene deletion mutants from the Madhani deletion collection were grown in YPD media, washed, and resuspended in PBS. Five 10-fold serial dilutions of each strain were spotted on YPD solid media, with the various additives as listed and incubated at 30°C for 48 h, unless otherwise stated. Strains were incubated at 39°C for 72 h before imaging. CFW: calcofluor white. (TIF) [file pgen.1006667.s005.tif]

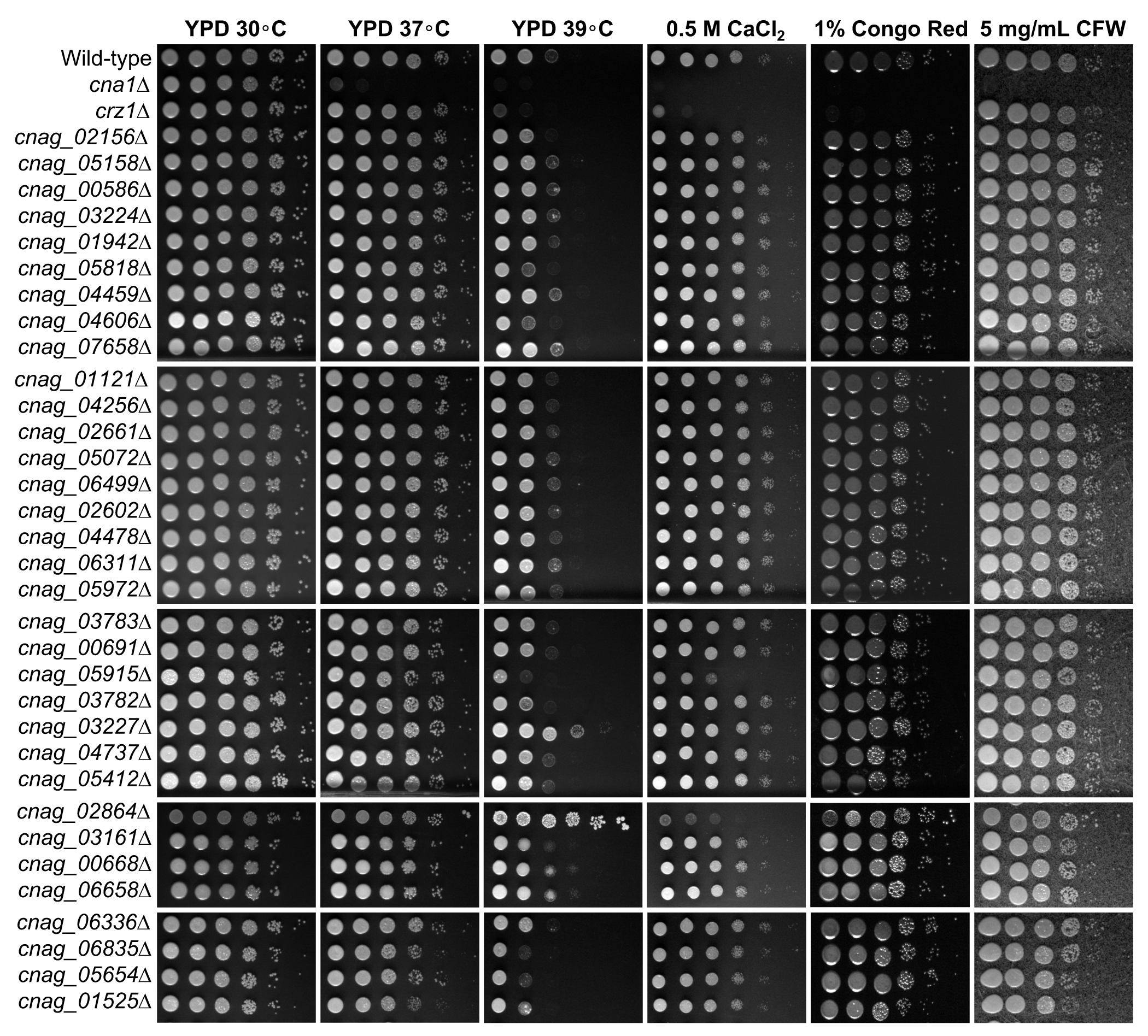

Supplement: S6 Fig — Wild-type (KN99α), cna1Δ (KK1), and crz1Δ (AFA1-4) strains and gene deletion mutants from the Madhani deletion collection were grown in YPD media, washed, and resuspended in PBS. Five 10-fold serial dilutions of each strain were spotted on YPD solid media, with the various additives as listed and incubated at 30°C for 48 h, unless otherwise stated. Strains were incubated at 39°C for 72 h before imaging. CFW: calcofluor white. (TIF) [file pgen.1006667.s006.tif]
