# Supplementary material for: Elucidation of the calcineurin-Crz1 stress response transcriptional network in the human fungal pathogen Cryptococcus neoformans
Source: PLoS Genet. 2017 Apr 4;13(4):e1006667. doi: 10.1371/journal.pgen.1006667 (PMC5380312; doi:10.1371/journal.pgen.1006667)
Supplement: S1 Table — Gene fold-change values from the cna1Δ and the crz1Δ mutant were compared against wild-type separately, and genes were deemed differentially expressed if the fold-change was ≥2-fold. Within each comparison, genes were organized in ascending log2FC values. Gene names and descriptions listed were identified using the FungiDB search portal; gene orthology was determined using the GO function. Log2FC = Log2 Fold change (DOCX) [file pgen.1006667.s007.docx]

**S1 Table: Genes differentially expressed in *cna1*Δ and *crz1*Δ mutants under non-induced conditions.**

| **Locus tag (CNAG)** | **Gene Name** | ***cna1*Δ log2FC** | ***crz1*Δ log2FC** | **Description** |
| --- | --- | --- | --- | --- |
| **Genes differentially expressed in both** ***cna1*Δ and *crz1*Δ** | | | | |
| 05158 |  | -1.20 | -1.49 |  |
| 05654 |  | -1.11 | -1.13 |  |
| 07498 |  | -1.01 | -1.60 | DUF567 domain-containing protein |
| 06347 |  | 2.36 | -1.17 |  |
| **Genes differentially expressed in** ***cna1*Δ** | | | | |
| 04796 | *CNA1* | -2.03 |  | serine/threonine-protein phosphatase 2B catalytic subunit A1 |
| 03538 |  | -1.28 |  |  |
| 06375 |  | -1.26 |  |  |
| 05331 |  | -1.25 |  |  |
| 03051 |  | -1.25 |  | Polyamine transporter |
| 02864 |  | -1.24 |  |  |
| 06203 |  | -1.21 |  |  |
| 05803 | *EXG1* | -1.18 |  | Exo-β-1,3-glucanase |
| 06598 |  | -1.13 |  |  |
| 06082 |  | -1.09 |  | Delayed-type hypersensitivity antigen |
| 01621 |  | -1.02 |  |  |
| 05239 |  | -1.02 |  | Cytoplasmic protein |
| 02722 |  | -1.01 |  | Aldose reductase |
| 06666 |  | 1.03 |  | Glycogen phosphorylase |
| 01704 |  | 1.04 |  | Serine/threonine protein kinase |
| 06009 |  | 1.05 |  | Cyclohydrolase |
| 02264 |  | 1.08 |  | AFG1-family ATPase |
| 04696 |  | 1.10 |  | DNA clamp loader |
| 04307 | *URO1* | 1.12 |  | Uricase |
| 01272 |  | 1.14 |  |  |
| 01949 |  | 1.16 |  | L-iditol 2-dehydrogenase |
| 02934 |  | 1.25 |  |  |
| 06918 |  | 1.37 |  |  |
| 05662 | *ITR4* | 1.40 |  | *Myo*-inositol transporter |
| 01223 |  | 1.42 |  |  |
| 00601 |  | 1.46 |  | Glycosyl hydrolase |
| 03238 |  | 1.79 |  | Dioxygenase subfamily protein |
| 01562 |  | 1.84 |  |  |
| **Genes differentially expressed in** ***crz1*Δ** | | | | |
| 00301 |  |  | -1.12 |  |
