# Supplementary material for: Elucidation of the calcineurin-Crz1 stress response transcriptional network in the human fungal pathogen Cryptococcus neoformans
Source: PLoS Genet. 2017 Apr 4;13(4):e1006667. doi: 10.1371/journal.pgen.1006667 (PMC5380312; doi:10.1371/journal.pgen.1006667)
Supplement: S2 Table — Gene fold-change values from the cna1Δ mutant were compared against wild-type and genes were deemed differentially expressed if the fold-change was ≥2-fold. Within each function classification, genes were organized in ascending log2FC values. Gene names and descriptions listed were identified using the FungiDB search portal; gene orthology was determined using the GO function. Log2FC = Log2 Fold change (DOCX) [file pgen.1006667.s008.docx]

**S2 Table: Genes regulated by calcineurin, independent of Crz1, under thermal stress.**

| **Locus Tag (CNAG)** | **Gene Name** | **Log2FC** | **Description** |
| --- | --- | --- | --- |
| 04796 | *CNA1* | -1.77 | Calcineurin A catalytic subunit |
| ***Transport*** | | | |
| 05387 |  | -1.91 | Galactose transporter |
| 00979 | *CTR4* | -1.80 | Copper uptake transporter |
| 03051 |  | -1.71 | Polyamine transporter |
| 05994 |  | -1.51 | Multidrug transporter |
| 01936 |  | -1.49 | Sugar transporter |
| 06020 | *SSU1* | -1.30 | Sulfite efflux pump |
| 01384 |  | -1.24 | Tartrate transporter |
| 06521 |  | -1.19 | Receptor/ Galactose-proton symporter |
| 01925 |  | -1.13 | Tartrate transporter |
| 05662 | *ITR4* | -1.13 | *Myo*-inositol transporter |
| 03824 | *MIR1* | -1.05 | Phosphate transport protein |
| 04015 |  | -1.04 | Amino acid transporter |
| 06561 |  | -1.02 | Allantoate transporter |
| 05993 |  | -1.02 | Transmembrane transporter Liz1p |
| 07641 |  | -1.02 | Monosaccharide transporter |
| 02586 |  | -1.00 | Sugar transporter |
| 03912 |  | 1.07 | Membrane protein |
| 00235 | *AMT1* | 1.07 | Ammonium transporter |
| 04414 | *FNX1* | 1.10 | Multidrug resistance protein |
| 01118 |  | 1.12 | Amino acid transporter |
| 03061 |  | 1.18 | Multiple drug resistance protein |
| 01055 |  | 1.18 | Phospholipid transporter |
| 03910 | *ITR6* | 1.19 | *Myo*-inositol transporter |
| 04758 | *AMT2* | 1.34 | Ammonium transporter |
| 00792 |  | 1.41 | ATP-binding cassette transporter |
| 05130 |  | 1.46 | Polyamide transporter |
| 07917 |  | 1.59 | Uracil transporter FurD |
| 06963 |  | 1.65 | Sugar transporter |
| 06610 |  | 1.67 | MSF transporter |
| 00796 | *MDR1* | 1.71 | Multidrug resistance protein 1 |
| 00904 | *AFLT* | 1.75 | Aflatoxin efflux pump |
| 03772 | *HXS1* | 1.85 | Glucose transporter |
| ***Oxidation-reduction*** | | | |
| 06169 |  | -1.91 | (R,R)-butanediol dehydrogenase |
| 00575 | *CAT3* | -1.66 | Catalase 3 |
| 04981 | *CAT1* | -1.63 | Catalase 1 |
| 02751 |  | -1.56 | Short-chain dehydrogenase |
| 06759 |  | -1.54 | Dehydrogenase |
| 02577 |  | -1.51 | Oxidioreductase |
| 01102 |  | -1.43 | Oxidoreductase |
| 04926 |  | -1.39 |  |
| 04351 |  | -1.32 | Methylmalonate-semialdehyde dehydrogenase |
| 04112 |  | -1.27 | Oxidoreductase |
| 01464 | *FHB1* | -1.24 | Flavohemoglobin |
| 04326 |  | -1.24 | Malate dehydrogenase |
| 00115 |  | -1.16 | Chlorophyll synthesis pathway protein BchC |
| 05299 |  | -1.13 | Oxidoreductase |
| 03865 |  | -1.11 | Cytoplasmic protein |
| 03874 |  | -1.11 | Oxidoreductase |
| 06081 |  | -1.11 | Glucose oxidase |
| 03555 |  | -1.09 | Acylglycerone-phosphate reductase |
| 01714 |  | -1.07 | Sulfonate dioxygenase |
| 05444 |  | -1.07 | NADPH dehydrogenase |
| 05644 |  | -1.03 | 2-nitropropane dioxygenase |
| 03194 |  | -1.04 | Saccharopine dehydrogenase |
| 04388 | *SOD2* | -1.00 | Mitochondrial manganese superoxide dismutase |
| 07626 |  | 1.02 | NAD-binding Rossmann fold oxidoreductase |
| 04313 |  | 1.07 | NADPH2 dehydrogenase |
| 01264 |  | 1.10 | Homoisocitrate dehydrogenase |
| 06876 |  | 1.13 | α-ketoglutarate-dependent taurine dioxygenase |
| 01627 |  | 1.14 | Amino oxidase |
| 04467 |  | 1.16 | Succinate-semialdehyde dehydrogenase |
| 05329 |  | 1.20 | *Myo*-inositol 2-dehydrogenase |
| 06250 |  | 1.29 |  |
| 06777 |  | 1.28 | Fructosyl amino acid oxidase |
| 05602 |  | 1.31 | 1-pyrroline-5-carboxylate dehydrogenase |
| 06628 |  | 1.35 | Aldehyde dehydrogenase |
| 01075 |  | 1.44 | Methylmalonate-semialdehyde dehydrogenase |
| 05115 |  | 1.53 | Sarcosine oxidase |
| ***Transcriptional regulation*** | | | |
| 03998 | *RLM1* | -1.29 | Transcriptional activator |
| 04512 |  | -1.11 | Transcription factor Spt20 homolog-like |
| 01847 |  | -1.08 |  |
| 04804 | *SRE1* | -1.05 | Sterol regulatory element-binding protein |
| 02435 | *BWC2* | -1.04 | White collar 2 transcription factor |
| 00332 | *SIP4* | 1.09 |  |
| 00627 | *SOX6* | 1.10 | Specific transcriptional repressor |
| 03086 | *FZC20* | 1.36 | Fungal specific transcription factor |
| 03849 | *ASG1* | 1.39 | Transcriptional regulator |
| 00791 | *HLH1* | 1.45 | Transcription factor bHLH041 |
| ***Protein kinases*** | | | |
| 00130 | *HRK1* | -1.10 | CAMK/CAMK1 protein kinase |
| 02531 | *CPK1* | 1.05 | CMGC/MAPK/ERK protein kinase |
| 02389 |  | 1.36 | AGC-group protein kinase |
| 06568 | *SKS1* | 1.38 | RAN protein kinase |
| ***DNA replication*** | | | |
| 05643 |  | -1.38 | DNA polymerase delta subunit 4 |
| 06440 |  | -1.17 | DNA dependent ATPase |
| 01013 | *SWD3* | -1.03 | Chromatin binding protein |
| 05374 | *CTF18* | 1.03 | Chromosome transmission fidelity protein 18 |
| 06745 |  | 1.05 | Histone H3 |
| 03341 | *MCM2* | 1.09 | Minichromosome maintenance protein 2 |
| 04696 |  | 1.12 | DNA clamp loader |
| 06634 | *DPB2* | 1.12 | DNA polymerase epsilon subunit |
| 07909 | *DCM1* | 1.21 | Meiotic recombinaase Dmc1 |
| 06166 | *MPH1* | 1.34 | ATP-dependent DNA helicase MPH1 |
| 00550 | *MSH5* | 1.46 | DNA mismatch repair protein MSH5 |
| ***Sterol biosynthesis*** | | | |
| 00519 | *ERG3* | -1.67 | C-5 sterol desaturase |
| 01737 | *ERG25* | -1.39 | C-4 methyl sterol oxidase |
| 06829 | *ERG1* | -1.02 | Squalene monooxygenase |
| 06644 | *ERG5* | -1.00 | C-22 sterol desaturase |
| ***Stress response*** | | | |
| 03143 |  | -3.31 | Heat shock protein (12 kDa) |
| 01446 | *HSP12* | -1.61 | Heat shock protein (12 kDa) |
| 03228 |  | -1.59 | Universal stress protein |
| 06121 |  | -1.35 |  |
| ***Carbohydrate metabolism/energy*** | | | |
| 00826 |  | -1.98 | Glycerone kinase |
| 00984 |  | -1.76 | Glucose and ribitol dehydrogenase |
| 00866 |  | -1.75 | Transketolase |
| 02230 |  | -1.64 | Phosphoketolase |
| 01341 |  | -1.60 | Mannose-6-phosphate isomerase |
| 06291 | *FPD1* | -1.50 | Polysaccharide deacetylase |
| 00827 |  | -1.54 | Ribose 5-phosphate isomerase |
| 04025 |  | -1.53 | Transaldolase |
| 05383 |  | -1.51 | 3-dehydroshikimate dehydratase |
| 02986 | *YSA1* | -1.50 | ADP-ribose pyrophosphatase |
| 06868 |  | -1.50 | Phosphopyruvate hydratase |
| 04744 |  | -1.38 | Mannose-6-phosphate isomerase |
| 06931 |  | -1.30 | β-glucosidase |
| 06923 | *XFP2* | -1.24 | Xylulose 5-phosphate/fructose 6-posphate phosphoketolase |
| 03040 |  | -1.24 | Transketolase |
| 05652 |  | -1.04 | Cytoplasmic protein |
| 07445 |  | 1.08 | Transketolase |
| 04969 | *UGD1* | 1.08 | UDP-glucose 6-dehydrogenase |
| 05264 |  | 1.33 | Alpha-amylase AmyA |
| 05913 |  | 1.89 | Alpha-glucosidase |
| ***Cell wall and capsule biosynthesis*** | | | |
| 02850 |  | 1.02 | Glucan endo-1,3-α-glucosidase AGN1 |
| 06031 | *KRE63* | 1.06 | β-glucan synthase |
| ***Other functions*** | | | |
| 03759 |  | -3.31 | Conidiation-specific protein 6 |
| 06238 |  | -2.32 | Glutathione S-transferase |
| 01348 |  | -1.64 | Cyanate hydratase |
| 00834 |  | -1.62 | Phosphatidylserine decarboxylase |
| 03084 |  | -1.56 | Endoribonuclease L-PSP |
| 01954 |  | -1.42 | Aldo/keto reductase |
| 03958 |  | -1.41 |  |
| 05268 |  | -1.37 | N-acetyltransferase 9-like protein |
| 06576 | *CAR1* | -1.37 | cAMP-regulated gene 1 |
| 02182 | *GRE2* | -1.30 | D-lactaldehyde dehydrogenase |
| 05449 | *CMT1* | -1.25 | Copper metallothionein |
| 02781 |  | -1.25 | Dihydrodipicolinate synthetase family protein |
| 04656 |  | -1.24 | Arginyl-tRNA-protein transferase |
| 04291 |  | -1.23 | Glycosyl-hydrolase |
| 04067 |  | -1.22 | Haloacid dehalogenase, type II |
| 04890 |  | -1.20 |  |
| 05386 |  | -1.20 | Glutamate 5-kinase |
| 00036 |  | -1.17 | Sec14 cytosolic factor |
| 06500 | *ARG82* | -1.16 | Arginine metabolism transcriptional control protein |
| 02768 |  | -1.16 |  |
| 00264 |  | -1.16 | Nuclease 1 |
| 00522 |  | -1.16 | C2 domain containing protein |
| 02417 |  | -1.15 | Lipase/esterase family protein |
| 00800 |  | -1.11 | Nicotinamidase |
| 04314 |  | -1.11 | NAD+ kinase |
| 02226 |  | -1.10 |  |
| 02722 |  | -1.10 | Aldose reductase |
| 03069 |  | -1.10 | Hydrolase |
| 04440 |  | -1.07 | Thiamine pyrophosphokinase |
| 06901 | *ALG7* | -1.07 | UDP-N-acetyl-glucosamine-1-p transferase |
| 04274 |  | -1.06 | Acyl-CoA thioesterase |
| 06782 | *MNN5* | -1.06 | Alpha-1,2-mannosyltransferase |
| 03070 |  | -1.04 | Compass compoment swd2 |
| 04659 |  | -1.03 | Pyruvate decarboxylase |
| 01521 |  | -1.03 | Metallo-beta-lactmase |
| 00254 |  | -1.01 | NADH dehydrogenase |
| 03698 |  | -1.01 | E3 ubiquitin-protein ligase UBR7 |
| 03738 |  | -1.01 | Pantetheine-phosphate adenylyltransferase |
| 02427 |  | -1.00 |  |
| 02725 |  | 1.00 | Proteasome subunit |
| 00062 |  | 1.01 | 26S proteasome non-ATPase regulatory subunit 3 |
| 01726 |  | 1.01 | FACT complex subunit SPT16 |
| 01442 |  | 1.05 | Cysteine desulfhydrase |
| 01148 |  | 1.03 | Peptidyl prolyl cis-trans isomerase |
| 07779 |  | 1.03 | D-glycerate 3-kinase |
| 01577 |  | 1.07 | Glutamate dehydrogenase |
| 00309 |  | 1.08 | Membrane protein |
| 04307 | *URO1* | 1.08 | Uricase |
| 03540 |  | 1.09 | Aspartyl/glutamyl-tRNA amidotransferase subunit B |
| 02309 |  | 1.11 | FK506-binding protein 2 |
| 04269 |  | 1.15 | Leucyl aminopeptidse |
| 02099 |  | 1.17 | Fatty acid synthase beta subunit |
| 06141 |  | 1.17 | dUTP diphosphatase |
| 00176 |  | 1.21 | Glutamate carboxypeptide protein |
| 03949 |  | 1.25 | 4-nitrophenylphosphatase |
| 05653 |  | 1.25 | Malate synthase |
| 05548 |  | 1.26 |  |
| 01565 |  | 1.27 | Biotin-[acetyl-CoA-carboxylase] ligase |
| 03394 |  | 1.28 |  |
| 00919 |  | 1.32 | Carboxypeptidase D |
| 01026 |  | 1.37 | Gamma-glutamyltranspeptidase 1 |
| 00758 |  | 1.41 | Regulatory protein ral2 |
| 02100 |  | 1.43 | Fatty acid synthase subunit alpha |
| 00141 |  | 1.43 | Monooxygenase |
| 01879 |  | 1.48 | Carboxypeptidase Y |
| 05759 |  | 1.63 | Acetyl CoA carboxylase |
| 00236 |  | 1.65 | 8-amino-7-oxononanoatesynthase |
| 01004 |  | 1.69 |  |
| 00601 | *CEL1* | 1.79 | Glycosyl hydrolase |
| 00164 |  | 2.00 |  |
| ***Unknown functions*** | | | |
| 05939 |  | -2.22 |  |
| 07939 |  | -2.13 |  |
| 06396 |  | -2.09 |  |
| 03492 |  | -2.09 |  |
| 02591 |  | -2.08 |  |
| 02070 |  | -2.07 |  |
| 02899 |  | -2.07 |  |
| 03679 |  | -1.95 |  |
| 00091 |  | -1.93 |  |
| 03142 |  | -1.93 |  |
| 00848 |  | -1.91 |  |
| 06109 |  | -1.88 |  |
| 03873 |  | -1.83 |  |
| 04139 |  | -1.79 |  |
| 04043 |  | -1.79 | DUF636 domain-containing protein |
| 01588 |  | -1.79 | Plasma membrane proteolipid 3 |
| 04943 |  | -1.78 |  |
| 04691 |  | -1.77 |  |
| 01735 |  | -1.73 |  |
| 04322 |  | -1.72 |  |
| 03058 |  | -1.70 | Hmp1 protein |
| 01090 |  | -1.67 |  |
| 04106 |  | -1.66 |  |
| 01921 |  | -1.65 |  |
| 07870 |  | -1.63 |  |
| 06718 |  | -1.61 |  |
| 03495 |  | -1.60 |  |
| 01751 |  | -1.57 |  |
| 04585 |  | -1.55 |  |
| 03408 |  | -1.52 |  |
| 06286 |  | -1.44 |  |
| 06493 |  | -1.44 |  |
| 02524 |  | -1.44 |  |
| 02118 |  | -1.43 |  |
| 04163 |  | -1.43 |  |
| 06302 |  | -1.43 | SnoaL domain |
| 00813 |  | -1.42 |  |
| 05666 |  | -1.41 |  |
| 00011 |  | -1.41 |  |
| 05167 |  | -1.40 |  |
| 06668 |  | -1.40 |  |
| 00807 |  | -1.39 |  |
| 03538 |  | -1.38 |  |
| 04206 |  | -1.38 | DUF636 domain-containing protein |
| 00093 |  | -1.37 |  |
| 02362 |  | -1.36 |  |
| 03566 |  | -1.35 |  |
| 01585 |  | -1.35 |  |
| 07666 |  | -1.35 | Cysteine-rich secretory protein family |
| 01816 |  | -1.34 |  |
| 06267 |  | -1.34 |  |
| 02043 |  | -1.33 |  |
| 01368 |  | -1.32 |  |
| 04938 |  | -1.32 |  |
| 01803 |  | -1.31 |  |
| 03232 |  | -1.31 | Lactamase |
| 03268 |  | -1.31 |  |
| 06395 |  | -1.29 |  |
| 01939 |  | -1.29 |  |
| 04167 |  | -1.28 |  |
| 00139 |  | -1.27 |  |
| 07673 |  | -1.27 |  |
| 05891 |  | -1.26 | TDG/mug DNA glycosylase |
| 04386 |  | -1.25 |  |
| 04880 |  | -1.25 |  |
| 07574 |  | -1.25 |  |
| 07450 |  | -1.25 |  |
| 02297 |  | -1.24 |  |
| 00595 |  | -1.24 |  |
| 05399 |  | -1.23 |  |
| 03688 |  | -1.23 |  |
| 05097 |  | -1.23 |  |
| 02347 |  | -1.22 |  |
| 03704 |  | -1.21 |  |
| 00465 |  | -1.21 |  |
| 04076 |  | -1.21 |  |
| 02058 |  | -1.20 |  |
| 01052 |  | -1.20 |  |
| 02129 |  | -1.20 |  |
| 03252 |  | -1.19 |  |
| 00075 |  | -1.19 |  |
| 00961 |  | -1.18 |  |
| 06805 |  | -1.17 |  |
| 03705 |  | -1.17 |  |
| 01691 |  | -1.17 |  |
| 03352 |  | -1.16 |  |
| 06075 |  | -1.15 |  |
| 05608 |  | -1.15 |  |
| 06800 |  | -1.15 |  |
| 07511 |  | -1.14 | Nucleolar protein 15 |
| 01643 |  | -1.13 |  |
| 07733 |  | -1.13 |  |
| 04766 |  | -1.13 |  |
| 04154 |  | -1.13 |  |
| 01621 |  | -1.12 |  |
| 02570 |  | -1.12 |  |
| 06602 |  | -1.12 | Cysteine-type peptidase |
| 01753 |  | -1.11 |  |
| 03667 |  | -1.11 |  |
| 05686 |  | -1.10 |  |
| 06297 |  | -1.10 |  |
| 04027 |  | -1.10 |  |
| 07972 |  | -1.10 |  |
| 01031 |  | -1.10 |  |
| 03454 |  | -1.10 |  |
| 06388 |  | -1.09 |  |
| 01417 |  | -1.09 |  |
| 03991 |  | -1.09 | Integral membrane protein |
| 01476 |  | -1.08 |  |
| 00054 |  | -1.08 |  |
| 00849 |  | -1.07 |  |
| 06245 |  | -1.07 |  |
| 02102 |  | -1.07 |  |
| 02253 |  | -1.07 |  |
| 01743 |  | -1.07 |  |
| 05682 |  | -1.07 |  |
| 01215 |  | -1.06 |  |
| 00074 | *PNS1* | -1.06 | Protein PNS1 |
| 03082 |  | -1.05 | Cupin domain-containing protein |
| 04466 |  | -1.05 |  |
| 04892 |  | -1.05 |  |
| 00454 |  | -1.05 |  |
| 06298 |  | -1.05 |  |
| 06207 |  | -1.05 |  |
| 07737 |  | -1.05 |  |
| 03002 |  | -1.05 |  |
| 02706 |  | -1.04 |  |
| 06516 |  | -1.04 |  |
| 00932 |  | -1.04 |  |
| 05520 |  | -1.03 |  |
| 00851 |  | -1.03 |  |
| 03685 |  | -1.02 |  |
| 02188 |  | -1.02 |  |
| 05937 |  | -1.02 |  |
| 07826 |  | -1.02 |  |
| 02444 |  | -1.02 |  |
| 00453 |  | -1.02 | Mitochondrial protein |
| 03455 |  | -1.01 |  |
| 01275 |  | -1.01 |  |
| 08019 |  | -1.01 |  |
| 04396 |  | -1.01 |  |
| 02422 |  | -1.01 |  |
| 03771 |  | -1.01 | DNA binding protein Ncp1 |
| 06082 |  | -1.01 | Delayed-type hypersensitivity antigen |
| 06827 |  | -1.00 |  |
| 02424 |  | -1.00 |  |
| 01493 |  | -1.00 |  |
| 02016 |  | -1.00 | DUF1479 domain-containing protein |
| 06515 |  | -1.00 |  |
| 01656 |  | 1.00 |  |
| 00936 |  | 1.00 | Lipid particle protein |
| 01538 |  | 1.02 |  |
| 07700 |  | 1.02 |  |
| 00149 |  | 1.03 |  |
| 01411 |  | 1.03 |  |
| 02735 |  | 1.03 |  |
| 01749 |  | 1.03 |  |
| 04261 |  | 1.03 |  |
| 05227 |  | 1.04 |  |
| 07463 |  | 1.04 | Separase |
| 01995 |  | 1.05 |  |
| 07479 |  | 1.06 |  |
| 04963 |  | 1.06 |  |
| 05738 |  | 1.08 |  |
| 06583 |  | 1.08 |  |
| 06608 |  | 1.09 |  |
| 01864 |  | 1.09 |  |
| 07415 |  | 1.10 |  |
| 03856 |  | 1.10 |  |
| 01180 |  | 1.11 |  |
| 06624 |  | 1.12 |  |
| 05515 |  | 1.13 |  |
| 01977 |  | 1.16 |  |
| 07548 |  | 1.16 | Cytoplasmic protein |
| 04707 |  | 1.18 |  |
| 06567 |  | 1.19 |  |
| 07945 |  | 1.19 |  |
| 00699 |  | 1.19 | Transmembrane receptor |
| 02882 |  | 1.20 |  |
| 06356 |  | 1.24 | Mitochondrial protein |
| 00194 |  | 1.25 |  |
| 01993 |  | 1.25 |  |
| 02192 |  | 1.25 |  |
| 01562 |  | 1.26 |  |
| 00177 |  | 1.27 |  |
| 01944 |  | 1.27 |  |
| 05514 |  | 1.28 |  |
| 05637 |  | 1.30 |  |
| 05457 |  | 1.31 | Pentatricopeptide repeat protein |
| 01244 |  | 1.34 |  |
| 06690 |  | 1.35 |  |
| 00190 |  | 1.38 |  |
| 01443 |  | 1.38 |  |
| 02705 |  | 1.48 |  |
| 05129 |  | 1.49 |  |
| 00798 |  | 1.52 |  |
| 00474 |  | 1.62 |  |
| 06000 |  | 1.79 | Glycoprotein |
| 02759 |  | 2.40 |  |
|  |  |  |  |
