# Supplementary material for: Elucidation of the calcineurin-Crz1 stress response transcriptional network in the human fungal pathogen Cryptococcus neoformans
Source: PLoS Genet. 2017 Apr 4;13(4):e1006667. doi: 10.1371/journal.pgen.1006667 (PMC5380312; doi:10.1371/journal.pgen.1006667)
Supplement: S3 Table — Gene fold-change values from the crz1Δ mutant were compared against wild-type and genes were deemed differentially expressed if the fold-change was ≥2-fold. Within each function classification, genes were organized in ascending log2FC values. Gene names and descriptions listed were identified using the FungiDB search portal; gene orthology was determined using the GO function. Log2FC = Log2 Fold change (DOCX) [file pgen.1006667.s009.docx]

**S3 Table: Genes differentially regulated by Crz1 under thermal stress, in a calcineurin-independent manner.**

| **Locus Tag**  **(CNAG)** | **Gene Name** | **Log2FC** | **Description** |  |
| --- | --- | --- | --- | --- |
| 00156 | *SP1/CRZ1* | -1.29 | Zinc finger transcription factor |  |
| ***Cell wall synthesis*** | | | | |
| 06508 | *FKS1* | -1.07 | 1,3-Beta-D-glucan synthase |  |
| 07499 | *CHS8* | -1.02 | Chitin synthase |  |
| ***Vesicle-mediated transport*** | | | | |
| 05277 |  | -1.47 | Vesicle-associated membrane protein |  |
| 05615 |  | -1.17 | Syntaxin1B/2/3 |  |
| ***Transport*** |  |  |  |  |
| 06242 | *CFT1* | 1.00 | Major iron permease |  |
| 01683 | *STL1* | 1.02 | Putative monosaccharide transporter |  |
| 06290 | *SNF3* | 1.03 | High-affinity glucose transporter |  |
| 03426 | *GMT2* | 1.04 | GDP-mannose transporter |  |
| 00869 | *PDR5* | 1.24 | ATP-binding cassette transporter |  |
| 06503 |  | 1.34 | Uridine permease |  |
| ***Carbohydrate metabolism*** | | | | |
| 05411 |  | -1.29 | Endoglucase |  |
| 03146 |  | -1.07 | Alpha-amylase |  |
| 02588 |  | 1.01 | Avenacinase |  |
| 06936 |  | 1.03 | Beta-glucosidase |  |
| ***Oxidation-reduction*** | | | | |
| 00692 |  | -1.18 | FAD dependent oxidoreductase |  |
| 07770 | *FRE1* | -1.04 | Ferric reductase |  |
| 02958 | *CFO2* | 1.01 | Ferroxidase |  |
| 06241 | *CFO1* | 1.12 | Ferroxidase/laccase |  |
| ***Protein kinases*** | | | | |
| 04514 | *MPK1* | -1.83 | Mitogen-activating protein kinase |  |
| 02194 |  | -1.62 | AGC/NDR protein kinase |  |
| 01704 |  | -1.46 | Serine/threonine protein kinase |  |
| ***Other functions*** | | | | |
| 04380 |  | -1.54 | Peptidase |  |
| 01539 |  | -1.48 | *Myo*-inositol-1-phosphate synthase |  |
| 06016 | *CAP6* | -1.15 | Alpha-1,3-mannosyltransferase |  |
| 04350 |  | -1.11 | Glycerophosphoryl diester phosphodiesterase |  |
| 02458 |  | -1.01 | GTPase activating protein |  |
| ***Unknown functions*** | | | | |
| 05302 |  | -2.12 |  |  |
| 02008 | *OVA2* | -1.87 |  |  |
| 04280 |  | -1.83 |  |  |
| 04752 |  | -1.69 |  |  |
| 01854 |  | -1.61 | Heparinase II/III family protein |  |
| 07638 |  | -1.57 |  |  |
| 01207 |  | -1.47 |  |  |
| 06507 |  | -1.44 |  |  |
| 07873 |  | -1.35 | MIPC synthase |  |
| 01796 |  | -1.43 |  |  |
| 06473 |  | -1.40 |  |  |
| 06973 |  | -135 |  |  |
| 02114 |  | -1.32 |  |  |
| 07723 |  | -1.31 |  |  |
| 01131 |  | -1.25 |  |  |
| 06060 |  | -1.23 |  |  |
| 06347 | *BLP2* | -1.20 | pr4/barwin domain protein |  |
| 03152 |  | -1.17 | Calcium-binding protein |  |
| 02585 |  | -1.17 |  |  |
| 03857 |  | -1.16 |  |  |
| 04171 |  | -1.14 |  |  |
| 02169 |  | -1.11 |  |  |
| 00261 |  | -1.09 |  |  |
| 05864 |  | -1.08 |  |  |
| 00274 |  | -1.06 |  |  |
| 06943 |  | -1.01 |  |  |
| 00349 |  | 1.02 |  |  |
| 01574 |  | 1.06 |  |  |
| 01070 |  | 1.08 | Class II aldolase/adducing family protein |  |
| 04355 |  | 1.17 |  |  |
| 07388 |  | 1.35 |  |  |
| 01873 |  | 1.48 |  |  |
