# Supplementary material for: Elucidation of the calcineurin-Crz1 stress response transcriptional network in the human fungal pathogen Cryptococcus neoformans
Source: PLoS Genet. 2017 Apr 4;13(4):e1006667. doi: 10.1371/journal.pgen.1006667 (PMC5380312; doi:10.1371/journal.pgen.1006667)
Supplement: S4 Table — Genes are deemed differentially expressed if the fold-change was ≥2-fold. Within each classification, genes were organized in ascending log2FC values. Gene names and descriptions listed were identified using the FungiDB search portal; gene orthology was determined using the GO function. Log2FC = Log2 Fold change (DOCX) [file pgen.1006667.s010.docx]

**S4 Table: 102 genes are differentially expressed in *cna1*Δ and *crz1*Δ mutants under thermal stress.**

| Locus tag (CNAG) | **Gene Name** | ***cna1*Δ log2FC** | ***crz1*Δ log2FC** | **Description** |  |
| --- | --- | --- | --- | --- | --- |
| *Cell wall synthesis* | | | | | |
| 01230 | *CDA2* | -2.47 | -2.13 | Chitin deactylase 2 |  |
| 06835 | *KRE61* | -2.36 | -1.47 | Glucosidase |  |
| 00546 | *CHS6* | -2.17 | -2.58 | Chitin synthase 6 |  |
| 05803 | *EXG1* | -2.09 | -1.38 | Exo-β-1,3-glucanase |  |
| 05818 | *CHS5* | -1.91 | -2.99 | Chitin synthase 5 |  |
| 06336 | *BGL2* | -1.78 | -2.22 | Glucan 1,3 β-glucosidase |  |
| 06501 | *GAS1* | -1.75 | -2.29 | 1,3-β-glucanosyltransferase |  |
| 05458 |  | -1.68 | -1.33 | Endo-1,3(4)-β-glucanase |  |
| 05138 | *SPR1* | -1.61 | -1.85 | Exo-β-1,3-glucanase |  |
| 03412 | *CTS1* | -1.30 | -1.09 | Chitinase 1 |  |
| 02217 | *CHS7* | -1.26 | -1.70 | Chitin synthase 7 |  |
| 00663 |  | -1.18 | -1.17 |  |  |
| *Ion and small molecule transport* | | | | | |
| 03135 |  | -1.33 | -1.82 | Magnesium transporter NIPA2 |  |
| 03242 | *RIM2* | -1.25 | -1.02 | Peroxisomal membrane protein |  |
| 00025 | *VCX1* | -1.14 | -1.09 | Calcium ion transporter |  |
| 01232 | *PMC1* | -1.09 | -1.85 | Calcium-transporting ATPase |  |
| 05632 |  | -1.85 | -1.34 | Cation-independent mannose-6-phosphate receptor |  |
| *Pheromone related* | | | | | |
| 07406 | *MFα* | -1.49 | -1.56 | Pheromone alpha |  |
| 07407 | *MFα3* | -1.11 | -1.31 | Fungal mating-type pheromone |  |
| *Degradative enzymes* | | | | | |
| 00407 |  | -3.42 | -2.66 | Glyoxal oxidase |  |
| 02030 |  | -1.95 | -2.21 | Glyoxal oxidase |  |
| 03464 | *LAC2* | -1.71 | -1.75 | Laccase |  |
| 05731 |  | -1.24 | -1.77 | Glyoxal oxidase |  |
| *Signaling related* | | | | | |
| 02415 |  | -1.10 | -1.62 | Annexin XIV |  |
| 03316 | *RDI1* | -1.02 | -1.86 | Rho GDP-dissociation inhibitor 1 |  |
| 04634 |  | -1.02 | -1.04 |  |  |
| *Transcriptional regulation* | | | | | |
| 07725 | *ROX1* | -2.94 | -3.42 | Specific transcriptional repressor |  |
| *Other functions* | | | | | |
| 06658 |  | -1.62 | -1.82 | Rhomboid family membrane protein |  |
| 05889 |  | -1.61 | -1.15 | SH3YL1 protein |  |
| 05521 |  | -1.42 | -1.09 | Aldose reductase |  |
| 05072 |  | -1.32 | -1.32 | Endopeptidase |  |
| 06748 | *UTP7* | -1.26 | -1.55 | U3 snoRNA-associated protein 7 |  |
| 03463 | *LSP1* | -1.19 | -1.19 | Sphingolipid long chain base-responsive protein |  |
| 06499 |  | -1.17 | -1.32 | Ppapdc1 protein |  |
| 02602 |  | -1.16 | -1.03 | Flavonol synthase |  |
| 03019 |  | -1.12 | -1.26 | Long-chain acyl-CoA synthase |  |
| 04016 |  | -1.09 | -1.35 |  |  |
| 03599 |  | 1.13 | 1.08 | Mandelate racemase/muconate lactonizing enzyme |  |
| 04523 |  | 1.60 | 1.37 | Glyceraldehyde 3-phosphate dehydrogenase |  |
| *Unknown function* | | | | | |
| 00588 |  | -5.25 | -4.72 | Ricin-type beta-trefoil lectin domain |  |
| 04891 |  | -4.45 | -4.12 | Ricin-type beta-trefoil lectin domain |  |
| 02864 |  | -3.83 | -3.34 |  |  |
| 03223 |  | -3.47 | -4.16 | Ser-Thr-rich glycosyl-phosphatidyl-inositol-anchored membrane family |  |
| 02526 |  | -3.41 | -2.38 |  |  |
| 04903 |  | -3.18 | -1.13 |  |  |
| 02685 |  | -3.10 | -1.93 | Fasciclin domain |  |
| 05641 |  | -3.04 | -2.08 |  |  |
| 01081 |  | -2.98 | -2.43 | NADH(P)-binding domain |  |
| 02156 |  | -2.87 | -3.14 |  |  |
| 05916 |  | -2.77 | -2.40 |  |  |
| 05158 |  | -2.74 | -3.18 |  |  |
| 07981 |  | -2.57 | -1.39 |  |  |
| 01506 |  | -2.55 | -3.45 |  |  |
| 00587 |  | -2.53 | -2.49 |  |  |
| 03007 |  | -2.45 | -1.50 |  |  |
| 05660 |  | -2.40 | -1.49 |  |  |
| 05654 |  | -2.32 | -2.19 | SUR7/PalI family domain |  |
| 00586 |  | -2.26 | -1.86 |  |  |
| 03783 |  | -2.25 | -2.04 | ubiE/COQ5 methyltransferase family |  |
| 03224 |  | -2.24 | -2.04 |  |  |
| 00691 |  | -2.20 | -2.91 |  |  |
| 05915 |  | -2.18 | -1.77 |  |  |
| 07498 |  | -2.10 | -3.17 | DUF567 domain containing protein |  |
| 06863 |  | -2.09 | -1.36 |  |  |
| 03782 |  | -2.09 | -1.58 |  |  |
| 01942 |  | -1.98 | -1.36 |  |  |
| 03154 |  | -1.96 | -2.80 |  |  |
| 05448 |  | -1.86 | -1.05 |  |  |
| 00485 |  | -1.82 | -1.38 |  |  |
| 03227 |  | -1.75 | -1.01 | Avr1-like family domain |  |
| 04459 |  | -1.74 | -1.34 |  |  |
| 04606 |  | -1.67 | -1.54 |  |  |
| 07658 |  | -1.66 | -1.59 |  |  |
| 01121 |  | -1.65 | -1.63 |  |  |
| 00301 |  | -1.61 | -2.23 |  |  |
| 04094 |  | -1.58 | -1.06 |  |  |
| 02510 |  | -1.57 | -2.33 |  |  |
| 04256 |  | -1.53 | -1.22 | Zinc finger with UFM1-specific peptidase domain |  |
| 01525 |  | -1.53 | -1.56 |  |  |
| 01272 |  | -1.51 | -2.90 |  |  |
| 04737 |  | -1.50 | -1.09 |  |  |
| 05089 |  | -1.49 | -1.15 |  |  |
| 03047 |  | -1.46 | -1.28 | Organic solute transporter Ostalpha domain |  |
| 07943 |  | -1.45 | -1.06 | DUF1183 domain |  |
| 02661 |  | -1.40 | -1.95 | Glycosyl hydrolase catalytic core |  |
| 06149 |  | -1.40 | -1.48 |  |  |
| 05159 |  | -1.39 | -1.74 |  |  |
| 04681 |  | -1.33 | -1.94 |  |  |
| 03161 |  | -1.32 | -1.14 |  |  |
| 01087 |  | -1.31 | -1.37 | Zinc finger, C3HC4 type domain |  |
| 05412 |  | -1.25 | -2.95 |  |  |
| 00647 |  | -1.24 | -2.37 |  |  |
| 07549 |  | -1.23 | -1.53 | FYVE zinc finger |  |
| 00668 |  | -1.22 | -1.63 |  |  |
| 04874 |  | -1.20 | -1.45 | Probable endo-1,3(4)-beta-glucanase |  |
| 02125 |  | -1.11 | -1.07 |  |  |
| 03314 |  | -1.08 | 1-.45 | YTH domain family 2 |  |
| 01174 |  | -1.07 | -1.02 | Pheromone-regulated membrane protein 10 |  |
| 04478 |  | -1.07 | -1.10 |  |  |
| 06311 |  | -1.03 | -1.41 |  |  |
| 04634 |  | -1.02 | -1.04 |  |  |
| 05972 |  | 1.36 | 1.14 |  |  |
