# Supplementary material for: Elucidation of the calcineurin-Crz1 stress response transcriptional network in the human fungal pathogen Cryptococcus neoformans
Source: PLoS Genet. 2017 Apr 4;13(4):e1006667. doi: 10.1371/journal.pgen.1006667 (PMC5380312; doi:10.1371/journal.pgen.1006667)
Supplement: S5 Table — Orthologs were determined from the FungiBD database using OrthoMCL. Note that Afu3g10690 (pmcB) and Afu7g01030 (pmcC) are both homologs of CNAG_01232 and YGL006W (PMC1); both genes were downregulated under Ca2+ stress in A. fumigatus crzAΔ mutant. (DOCX) [file pgen.1006667.s011.docx]

**S5 Table. List of calcineurin-Crz1 regulated genes shared in common across species**

| **Calcineurin-dependent genes shared between *C. neoformans* and *S. cerevisiae*** | | |
| --- | --- | --- |
| ***C. neoformans*** | **Gene name** | ***S. cerevisiae*** |
| CNAG_01232 | Calcium-transporting ATPase | YGL006W (*PMC1*) |
| CNAG_02217 | Chitin synthase 7 | YNL192W (*CHS1*) |
| **Calcineurin-dependent genes shared between *C. neoformans* and *A. fumigatus*** | | |
| ***C. neoformans*** | **Gene name** | ***A. fumigatus*** |
| CNAG_00025 | Calcium ion transporter (*VCX1*) | Afu2g05325 |
| CNAG_01232 | Calcium-transporting ATPase (*PMC1*) | Afu3g10690 (*pmcB*) |
| CNAG_01232 | Calcium-transporting ATPase (*PMC1*) | Afu7g01030 (*pmcC*) |
| CNAG_02217 | Chitin synthase 7 (*CHS7*) | Afu2g01870 (*chsA*) |
| CNAG_03412 | Chitinase 1 (*CTS1*) | Afu7g08490 (*chiB1*) |
| CNAG_04737 | Hypothetical protein | Afu6g03450 |
| **Calcineurin-dependent genes shared between *S. cerevisiae* and *A. fumigatus*** | | |
| ***A. fumigatus*** | **Gene name** | ***S. cerevisiae*** |
| Afu3g10690 | Calcium-transporting ATPase (*pmcB*) | YGL006W (*PMC1*) |
| Afu7g01030 | Calcium-transporting ATPase (*pmcC*) | YGL006W (*PMC1*) |
| Afu2g01870 | Chitin synthase A (*chsA*) | YNL192W (*CHS1*) |
| Afu4g13270 | Unfolded protein response protein (*orm1*) | YLR350W (*ORM2*) |
