# Supplementary material for: Elucidation of the calcineurin-Crz1 stress response transcriptional network in the human fungal pathogen Cryptococcus neoformans
Source: PLoS Genet. 2017 Apr 4;13(4):e1006667. doi: 10.1371/journal.pgen.1006667 (PMC5380312; doi:10.1371/journal.pgen.1006667)
Supplement: S8 Table — (DOCX) [file pgen.1006667.s014.docx]

**S8 Table: List of fungal strains and plasmids used in the study.**

| **Name** | **Description** | **Reference** |
| --- | --- | --- |
| ***Fungal strains*** | | |
| KK1 | H99 MATα *cna1*Δ::*NAT* | This study |
| KK5 | H99 MATα *cna1*Δ::*NAT CNA1*::*NEO* | This study |
| KK8 | KN99 MAT**a** *cna1*Δ::*NEO* | This study |
| AFA3-3 | H99 MATα *crz1*Δ::*NAT* | This study |
| AFA1-4 | KN99 MAT**a** *crz1*Δ::*NAT* | This study |
| XW245 | H99 MATα *cna1*Δ::*NEO* *crz1*Δ::*NAT* | This study |
| AFA3-3-3 | H99 MATα *crz1*Δ::*NAT CRZ1*::NEO | This study |
| AFA3-3-17 | H99 MATα *crz1*Δ::*NAT CRZ1*::NEO | This study |
| ECt3 | H99 MATα *crz1*Δ::*NAT* + Crz1^WT^- mCherry-*NEO* | This study |
| ECt4 | H99 MATα *crz1*Δ::*NAT* + Crz1^WT^- mCherry-*NEO* | This study |
| ECt172 | H99 MATα *crz1*Δ::*NAT* + Crz1^WT^- mCherry-*NEO +* GFP-Nop1::*HYG* | This study |
| ECt54 | H99 MATα *crz1*Δ::*NAT +* GFP-Nop1::*HYG* | This study |
| ECt335 | H99 MATα *crz1*Δ::*NAT* + Crz1^S103,288, 329, 508, 569, 765, 810A^- mCherry-*NEO* + GFP-Nop1::*HYG* | This study |
| ECt375 | H99 MATα *crz1*Δ::*NAT* + Crz1^PMICIQΔ^- mCherry-*NEO* | This study |
| ECt394 | H99 MATα *crz1*Δ::*NAT* + Crz1^PMICIQΔ^- mCherry-*NEO* | This study |
| ECt386 | H99 MATα *crz1*Δ::*NAT* + Crz1^PMICIQΔ^- mCherry-*NEO +* GFP-Nop1::*HYG* | This study |
|  |  |  |
| ***Plasmids*** | | |
| pSDMA25 | Safe haven plasmid | Arras *et al.* 2015 |
| pEC13 | Crz1-mCherry fusion protein; *NEO* marker | This study |
| pSL04 | GPF-Nop1 fusion protein; *NAT* marker | Lee and Heitman 2012 |
| pEC28 | GPF-Nop1 fusion protein; *HYG* marker | This study |
| pEC99 | Crz1^PMICIQΔ^ – mCherry fusion protein; *NEO* marker | This study |
