# Supplementary material for: Elucidation of the calcineurin-Crz1 stress response transcriptional network in the human fungal pathogen Cryptococcus neoformans
Source: PLoS Genet. 2017 Apr 4;13(4):e1006667. doi: 10.1371/journal.pgen.1006667 (PMC5380312; doi:10.1371/journal.pgen.1006667)
Supplement: S9 Table — (DOCX) [file pgen.1006667.s015.docx]

**S9 Table: List of primers**

| **Primer Name (JOHE)** | **Description** | **Oligo Sequence** |
| --- | --- | --- |
| ***crz1* deletion construction primers** | | |
| 38810 | *crz1* deletion 5’ flank | TGATGATTTGTATTCGGGCG |
| 16870 | *crz1* deletion 5’ overlap | ctggccgtcgttttACAGGCTGTGCAAGATAACGGAGA |
| 16871 | *crz1* deletion 3’ overlap | gtcatagctgtttcctggCTTTCTTGCCATTCGTATGG |
| 38850 | *crz1* deletion 3’ flank | GGTTTAGGGGAGGTTGGGAT |
| 38858 | 5’ diagnostic primer | TTGCGCTGATCATTGGTACT |
| 38859 | 3’ diagnostic primer | TTTCGCCGCTCTGCCCTCTTA |
| ***CRZ1*-mCHERRY construction primers** | | |
| 39093 | Crz1 amplification | ATGGCAGATCCAGCCTCACCC |
| 39078 | Crz1 nested primer | GGGGAGCAGATTGTGTATCTT |
| 39079 | Crz1-mCherry overlap Reverse | ctcgcccttgctcaccatATCCTCTTCACTCGTTTCAC |
| 39080 | Crz1-mCherry overlap Forward | gagctcggtaccaagcttggTCGCCCGATGGTCATAG |
| 39082 | Crz1 nested primer | TGTGCTGAGGCTGTGTACCC |
| **Crz1^PMICIQΔ^ -mCHERRY construction primers** | |  |
| 42976 | PMICIQ motif deletion Forward | TATCGCACCCAATGCAGATGGGCCCAGTACACCGATAAGTGGAT |
| 42977 | PMICIQ motif deletion Reverse | ATCCACTTATCGGTGTACTGGGCCCATCTGCATTGGGTGCGATA |
| 43011 | Site-directed Gibson cloning Forward | ATACCCTGGTCATCCAATCATCG |
| **Sequencing primers** | | |
| 39136 | Primer 1 Forward | GTCCACTATGCCAGCGTTCA |
| 39137 | Primer 2 Reverse | CCTGCCTTCCTTCAATCCAC |
| 39138 | Primer 3 Forward | ATTCCCACCGTCAAATCCAG |
| 39139 | Primer 4 Reverse | GCTGATACAGTGCCGGACAA |
| 39140 | Primer 5 Forward | GCAGCAGACTGGACAAATGC |
| 39141 | Primer 6 Reverse | GGCGTCTGCTGCTCTTCATA |
| 39142 | Primer 7 Forward | GCCAGGCAACATGATTGTAAA |
| 40141 | Primer 8 Forward | CAATAATAAGCGATATTTAG |
| 40142 | Primer 9 Forward | GGACATTATCTTCAGATCAG |
| 40684 | Primer 10 Forward | GGTTCTGCGGGAATAGAT |
| 40685 | Primer 11 Forward | CACCTTTTCCTCAGCAATCA |
| 40686 | Primer 12 Reverse | TTGACGGTGGGAATAATC |
| 40687 | Primer 13 Reverse | CAGCTATTCGCAAGGTATC |
| 41346 | Primer 14 Forward | CGAGTGAAGAGGATATGGTG |
| 41347 | Primer 15 Reverse | TACATGAACTGAGGGGAC |
| 41348 | Primer 16 Forward | GCCCCGTAATGCAGAAGA |
| 41349 | Primer 17 Reverse | CGTTGTGGGAGGTGATGT |
| 41350 | Primer 18 Forward | CACCCCACTACCAAGCGAA |
| 41351 | Primer 19 Reverse | ATACTCTCAACACCAGCG |
| ***CRZ1* Complementation diagnostic primers** | | |
| 40956 | Previously described in Arras *et al.* 2015 | AACTATACGCAGCATGAACAT |
| 40957 | Previously described in Arras *et al.* 2015 | ACTTTCTACCTGGTCTGCTTT |
| 40958 | Previously described in Arras *et al.* 2015 | AATAAGGGCGACACGGAAATG |
| 41562 | Crz1 3’ Forward | ATGTGGATGTGGAGGAAGA |
| 41450 | Tandem array 5’ reverse | GCCCATAGACTTCAAATCATG |
| 41451 | Tandem array 3’ Forward | GGCAAGGGAACAGACAGATAC |
| **Real-Time qPCR validation primers** | | |
| 40361 | CNAG_06501 Forward | TCAATGCGGTCAGAGTGTATAGT |
| 40362 | CNAG_06501 Reverse | TCCGTTGAGTGGTAAGGAAAGA |
| 40365 | CNAG_01982 Forward | GTAGCATGGTGGATCTGGAAG |
| 40366 | CNAG_01982 Reverse | GGAGGAGAGAGCGAGTGAAATAG |
| 40369 | CNAG_04514 Forward | GAGCTCATGGAGGCTGATTTG |
| 40370 | CNAG_04514 Reverse | CTTGAGGCCACAGAGAGTTTG |
| 40375 | CNAG_02217 Forward | TCCTACAACCGAGAAACTGAACT |
| 40376 | CNAG_02217 Reverse | GCGCCAGAATTTAGACGCTTTG |
| 40377 | CNAG_06016 Forward | GATGGGTTGCGAGAGATATGAGT |
| 40378 | CNAG_06016 Reverse | CAGACGGTTGTAGCGTTTGAG |
| 40381 | CNAG_07545 Forward | GAAAGTGGCCGCGATACTATAAT |
| 40382 | CNAG_07545 Reverse | CCTGGACGATACTTTGACCCATAC |
| 40387 | CNAG_02527 Forward | GGTTGCTCTTACGGTCCTTATTC |
| 40388 | CNAG_02527 Reverse | TGGTGTAGAAAGGAGTGGCATATAG |
| 40528 | CNAG_01081 Forward | CTGGAAGGGTACAAAGCTAAGGA |
| 40529 | CNAG_01081 Reverse | TGCAACATACGAGTGCCAGTA |
| 40530 | CNAG_01121 Forward | CTTGAATTTGTCACTCGGTTCAGT |
| 40531 | CNAG_01121 Reverse | GGCGATGTCGATAGGGAAGTATT |
| 40534 | CNAG_00261 Forward | GGTTCCGCTCCTTACTATCTTGC |
| 40535 | CNAG_00261 Reverse | GGTACCAGTGCTGTCTGTAACC |
| 40538 | CNAG_06658 Forward | TGAGCAGGCTATTGAGAGGAA |
| 40539 | CNAG_06658 Reverse | TTGAAAGTTGGCCTGATAGCG |
| 16900 | CNAG_03224 Forward | TGGCACGAAGAACCACTTTC |
| 16901 | CNAG_03224 Reverse | CCTCTTTCACATCGGGCAAGATA |
| 16902 | CNAG_01230 Forward | CAGTACAGACATCAACACCGAG |
| 16903 | CNAG_01230 Reverse | TGGGCTTCTGTGTCATTGGA |
| 16906 | CNAG_03154 Forward | ACATGACTAGTCTCTATGTTACACC |
| 16907 | CNAG_03154 Reverse | CCAAGGATTCCACACGACTT |
| 16910 | CNAG_07498 Forward | TGCCTACGACCCTTGTTTT |
| 16911 | CNAG_07498 Reverse | GTCACAAAGTAAGTCTGTTTATCCC |
| 16914 | CNAG_00301 Forward | AGTGGTGCAGAAGTGAAGACG |
| 16915 | CNAG_00301 Reverse | ACACAGTAATCACCAGTTGAGGAA |
| 16918 | CNAG_01525 Forward | TAGAAAGTGGGTGGCAGAAAGA |
| 16919 | CNAG_01525 Reverse | CAAGGGTCAGTGGGTCGATA |
| 16920 | CNAG_03477 Forward | TTTAAAAGTGCCTTCTAAC |
| 16921 | CNAG_03477 Reverse | GAAGTCCTGGATCCCAAACCGG |
| 16926 | CNAG_07407 Forward | CCATCTTCACCACCTCCATTTCT |
| 16927 | CNAG_07407 Reverse | CCCTTTGTGTCATCGCCTAA |
| 16928 | CNAG_06973 Forward | ACTTGCAGGTAGCAAACTCTCC |
| 16929 | CNAG_06973 Reverse | GACGAGGTTATCTAGCCAAGCAT |
| 16932 | CNAG_01668 Forward | CATTCAAGCTCTTGACGGTAGGA |
| 16933 | CNAG_01668 Reverse | CTTCTTGAGGGTCAAGCGTAATC |
| 42947 | CNAG_00546 Forward | TCTTGCGGCTCTCAATTATG |
| 42948 | CNAG_00546 Reverse | CCTCAAGCTTATCGTCCACA |
| 43355 | CNAG_00588 Forward | CCCTAACAGCAACGACAAC |
| 43356 | CNAG_00588 Reverse | GTCCAGATCTTGACACCCT |
| **ChIP primers** | | |
| 41901 | CNAG_00407 Forward | AAATCGGTACGAGCTGGATG |
| 41902 | CNAG_00407 Reverse | GCAGTGGCTGTGTCTTTTGA |
| 42428 | CNAG_00588 Forward | GGGAGTGTCATGACGATGAAGT |
| 42429 | CNAG_00588 Reverse | GCAACTCGCCGACTCGTAAAT |
| 42432 | CNAG_04891 Forward | GGAGTGTTCAAAGGACAGAAGAT |
| 42433 | CNAG_04891 Reverse | ATATCTCGCAGCATGGAAGAAG |
|  |  |  |
